# Balancing Monitoring and Management in the Adaptive Management of an Invasive Species

**DOI:** 10.1002/ece3.71176

**Published:** 2025-04-01

**Authors:** Brielle K. Thompson, Julian D. Olden, Sarah J. Converse

**Affiliations:** ^1^ Washington Cooperative Fish and Wildlife Research Unit, Quantitative Ecology and Resource Management Program University of Washington Seattle Washington USA; ^2^ School of Aquatic and Fishery Sciences University of Washington Seattle Washington USA; ^3^ U.S. Geological Survey, Washington Cooperative Fish and Wildlife Research Unit, School of Environmental and Forest Sciences & School of Aquatic and Fishery Sciences University of Washington Seattle Washington USA

**Keywords:** adaptive management, community science data, invasive species, management strategy evaluation, monitoring

## Abstract

Efficient allocation of managers' limited resources is necessary to effectively control invasive species, but determining how to allocate effort between monitoring and management over space and time remains a challenge. In an adaptive management context, monitoring data are key for gaining knowledge and iteratively improving management, but monitoring costs money. Community science or other opportunistic monitoring data present an opportunity for managers to gain critical knowledge without a substantial reduction in management funds. We designed a management strategy evaluation to investigate optimal spatial allocation of resources to monitoring and management, while also exploring the potential for community science data to improve decision‐making, using adaptive management of invasive flowering rush ( *Butomus umbellatus* ) in the Columbia River, USA, as a case study. We evaluated management and monitoring alternatives under two invasion conditions, a well‐established invasion and an emerging invasion, for both risk‐neutral and risk‐averse decision makers. Simulations revealed that regardless of invasion condition or managers' risk tolerance, allocating effort outward from the estimated center of invasion (*Epicenter* prioritization) resulted in the lowest overall level of infestation at the end of management. This allocation outperformed alternatives in which management occurred in fixed areas (*Linear* prioritization) and alternatives that targeted patchily distributed areas with the highest estimated infestation level of the invasive species (*High invasion* prioritization). Additionally, management outcomes improved when more resources were allocated toward removal effort than monitoring effort, and the addition of community science data improved outcomes only under certain scenarios. Finally, actions that led to the best outcomes often did not produce the most accurate and precise estimates of parameters describing system function, emphasizing the importance of using value of information principles to guide monitoring. Our adaptive management approach is adaptable to many invasive species management contexts in which ongoing monitoring allows management strategies to be updated over time.

## Graphical abstract

Smart allocation of managers' limited resources is necessary to effectively control invasive species, but determining how to allocate effort between monitoring and management over space and time remains a challenge. We considered the question of optimal spatial allocation of resources to monitoring and management, while also considering the potential for community science data to improve decision making, using adaptive management of invasive flowering rush in the Columbia River, USA, as a case study.

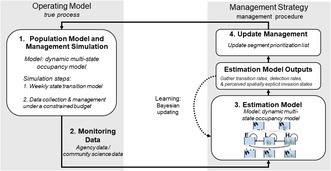

## 1 Introduction

Species invasions continue to increase globally (Sardain et al. 2019; Seebens et al. 2021), causing severe ecological and environmental damage (Doherty et al. 2016), resulting in over $400 billion per year in estimated damage worldwide (Bradshaw et al. 2024). Natural resource managers have opportunities to help thwart the negative consequences of invasions by implementing management using manual, chemical, or biocontrol efforts to suppress or eradicate invasive species. However, given that effective invasive species management approaches are context‐specific, questions regarding how managers can best allocate their limited resources over space and time to effectively monitor and manage invasive species are ubiquitous (Thompson et al. 2024; Van Poorten and Beck 2021).

Monitoring data are key to invasive species management (Pyšek and Richardson 2010). Monitoring to understand the distribution of an invasive species is critical for knowing where to manage (Lyons et al. 2008) and where to prioritize prevention efforts (Mandrak and Cudmore 2015). In addition, novel and valuable insights into invasive population dynamics can be gained from monitoring data (van Rees et al. 2022), including the factors driving the spread of invasive species and the effectiveness of management efforts for reducing distribution, abundance, or rates of spread. Species occupancy models are particularly useful for providing such insights. Occupancy models use detection/nondetection data to understand species occurrence, as well as the colonization and local extinction processes that drive species occurrence (MacKenzie et al. 2002, 2003).

Invasive species management efforts can benefit from a process known as adaptive management, in which monitoring data help to inform initial decisions and to update strategies based on information gained from the data (Walters 1986). Adaptive management differs from other approaches (e.g., trial‐and‐error or static management) by integrating a learning process. This process involves collecting monitoring data to allow decision makers to manage while learning, thereby reducing uncertainty and improving performance on management objectives over the long term (Lyons et al. 2008; Pepin et al. 2020). Adaptive management can be especially useful for invasive species management because uncertainties regarding species biology and the effectiveness of management actions are often substantial, especially for emerging invaders (Regan et al. 2011). However, monitoring can be costly and time intensive, and so it is necessary to carefully allocate limited resources to monitoring and management activities (Hauser and McCarthy 2009; Bogich et al. 2008; Baker et al. 2017).

Advancing methods to determine optimal allocation of resources towards monitoring and management is now considered paramount in invasive science (Yemshanov et al. 2019; Koch et al. 2020; Nguyen et al. 2024). Previous studies examining surveillance and control of invasive species have primarily focused on optimizing efforts under a single decision‐making entity that collects one type of data (Hauser and McCarthy 2009; Regan et al. 2011; Rout et al. 2017). In some cases, however, multiple entities—including the public—may contribute to monitoring (Crall et al. 2006, 2010; Graham et al. 2007). Community science programs may offer a cost‐effective approach for surveillance of invasive species and can contribute to public education and even novel detections of emerging invaders (Crall et al. 2013; Larson et al. 2020; Compagnone et al. 2023). Natural resource agencies that are actively controlling invasive species could collect monitoring data in known infestation areas while relying on community scientists to search for and report new species occurrences (sensu Sipe et al. 2023). Such data can provide critical information while not taking resources away from management, though potential challenges with community science data (e.g., species misidentification) also warrant consideration. Data integration techniques can be used to make more efficient and effective use of multiple data streams to estimate key parameters (Zipkin and Saunders 2018; Fletcher et al. 2019; Isaac et al. 2020) resulting in more accurate and precise parameter estimates across larger spatial extents (Dorazio 2014; Zipkin et al. 2021; Doser et al. 2022; Dimson et al. 2023).

Here, we evaluate the effectiveness of alternative monitoring and control (suppression) strategies for invasive species management, using invasive flowering rush ( *Butomus umbellatus* ) as an example. Flowering rush was introduced into North America in 1897 as an ornamental plant and was first found in the Columbia River Basin, USA, in 1949 (Anderson et al. 1974). This invasive plant can greatly alter ecosystems through sediment accretion and its ability to rapidly disperse, colonize, and produce dense stands of emergent and submerged growth. These stands can displace native vegetation and provide habitat for invasive predator species that impact salmon populations and, more broadly, riverine food webs (Cooper et al. 2008; Gunderson et al. 2016). We focus on flowering rush because its population is rapidly expanding in the Columbia River, increasing the urgency to evaluate and apply alternative management strategies for control (Columbia Basin Cooperative Weed Management Area 2019).

We developed spatial simulation models to explore alternative adaptive approaches for invasive flowering rush control at the leading edge of invasion, including different monitoring strategies. This allowed for the evaluation of whether management outcomes improve as a function of the amount or type (e.g., single‐ or multistate detection/nondetection data) of monitoring data. We simulated population dynamics based on existing knowledge of the ecology of flowering rush under various spatial allocation strategies, levels of investment (i.e., budget), invasion conditions (i.e., established and emerging invasions), and for both risk‐neutral and risk‐averse decision makers. The results from this study sought to answer two fundamental questions: (1) how much time should be allocated towards monitoring versus conducting management?; and (2) where should management and monitoring occur across a landscape? We used management strategy evaluation (Smith 1999)—a tool that, to the best of our knowledge, has not been used in invasive species management—to simulate the adaptive management process. Our framework is readily applicable to a variety of invasive species management challenges.

## 2 Methods

### 2.1 Management Context

**FIGURE 1:**
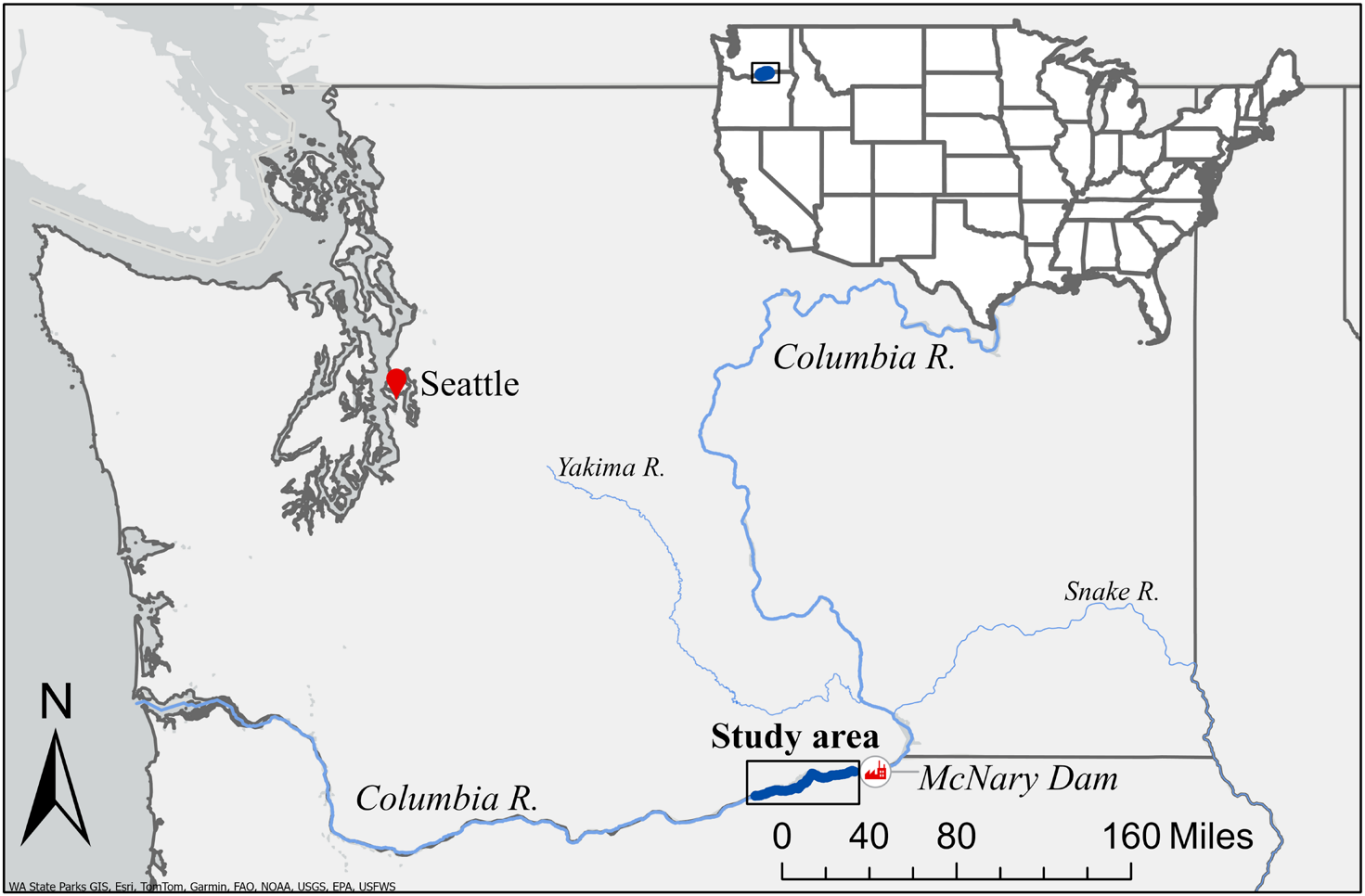
Map of the mainstem of the Columbia River, in light blue, and location of the study area, in dark blue and indicated with a box. The study area is a subset of the Columbia River that spans 40 miles (65 km) downstream of the McNary Dam.

Resource managers in the Columbia River Basin aim to manage flowering rush to reduce the negative ecological and economic effects (Columbia Basin CWMA 2019). For the past decade, flowering rush has been established in Washington State along the Columbia and Yakima Rivers. The current leading edge of flowering rush invasion in the mainstem of the Columbia River is projected to be downstream of McNary Dam (see fig. 15 in Columbia Basin CWMA 2019) and we focused our removal and monitoring in the 65‐km river section downstream of the dam (Figure 1). Informed by conversations with natural resource managers (see Acknowledgments) in the area, we assumed a management objective of minimizing the overall level of infestation of flowering rush across the study area; we refer to this as the “suppression” objective (see Section 2.6 for details). Natural resource managers are often concerned with the cost of conducting monitoring and management. We, therefore, developed alternatives involving different investment levels, defined by a fixed number of available labor hours per week (labor is the primary contributor to management costs).

We simulated management actions conducted at 1‐mile (1.6 km‐) long river management units, henceforth referred to as “segments” (Table 1). Following current management practices, we assumed that monitoring and management (via hand removal of flowering rush) would be conducted at the end of the growing season, for a 4‐week period starting August 1st, in each year over 10 years. We envisioned a situation in which, over this 4‐week period, segments along the river would be visited and some effort would be put into searching for the plant in those segments. Two independent individuals would search for a fixed number of hours, allowing for the estimation of system state using occupancy modeling (MacKenzie et al. 2002). If plants were found, a fixed number of hours of removal effort would be implemented. Then, the next segment would be visited, and so on, until the total effort available for the week was expended (or until all segments had been visited and searched).

**TABLE 1:** Strategy table depicting all individual actions that, in combination, constitute decision alternatives for the monitoring and management of flowering rush in the Columbia River.

Spatial priority	Target detection and eradication probabilities (*p*, ϵ)	Investment (hours/week)	Data
None: no segments are visited (*No removals*)	(0, 0)	None (0)	None
*Linear*: prioritized in order by space	(0.25, 0.5)	Low (20)	A
*High invasion*: segments are placed in one of three bins that describe invasion size, then segments are prioritized by highest estimated invasion bin and visited in order by distance	(0.25, 0.75)	Moderate (40)	A + C
*Epicenter*: prioritized segments outwards from the epicenter in the direction with the highest adjacent invasion, then segments were prioritized outwards in the other direction	(0.5, 0.5)	High (60)	
	(0.5, 0.75)		
	(0.75, 0.5)		
	(0.75, 0.75)		

*Note:* The alternatives were a combination of spatial priority (which segments were prioritized for visits); target detection, *p*, and eradication, ϵ, probabilities; management investment (maximum labor hours per week); and the type of monitoring data gathered (indicated with None if no monitoring data were collected, A if just agency data were collected, and A + C if agency and community science data were collected). In total, we simulated 61 alternatives—see text for details.

### 2.2 Management Strategy Evaluation

We developed an adaptive management framework, in which monitoring data are gathered to reduce uncertainties regarding system function and where management is adapted in response to learning (Walters 1986). We used management strategy evaluation (MSE) to simulate the process of managing and learning (Smith 1999). In comparison, stochastic dynamic programming (SDP) is the more typical approach for identifying optimal adaptive decisions (Walters and Hilborn 1978; Marescot et al. 2013). However, SDP algorithms are subject to the “curse of dimensionality,” in which addition of system complexity, for example, additional system states, increases the size of the optimization problem exponentially. Hence, we used MSE, which can incorporate a system model with more complex spatial–temporal biological processes than the models used in SDP (Mapstone et al. 2008; Bunnefeld et al. 2011; Pepin et al. 2022).

**FIGURE 2:**
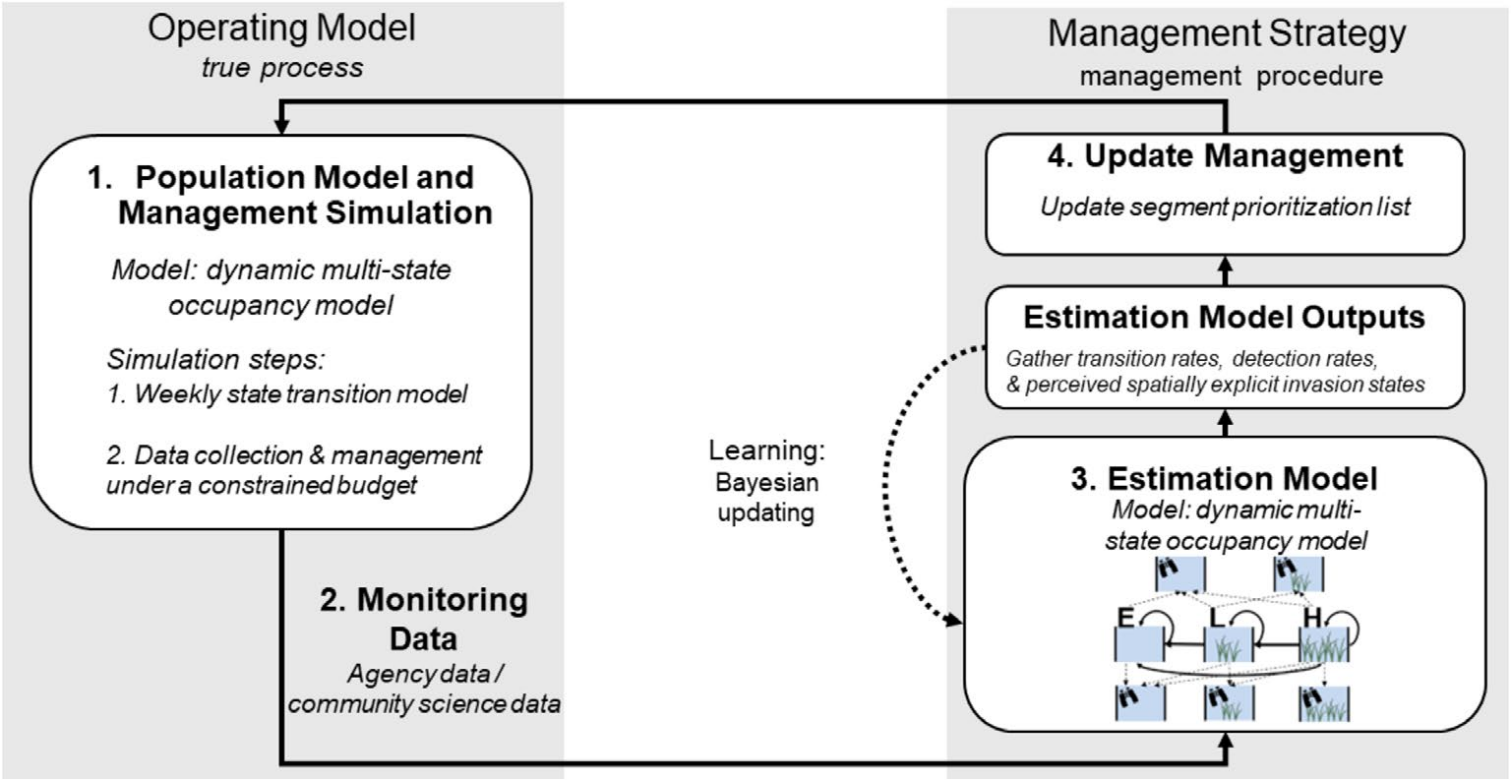
Depiction of our management strategy evaluation (MSE) approach to adaptive management: 1. Under multiple alternative management strategies, simulate realistic population dynamics and management of the species using an operating model based on distributions of parameter values representing the current state of knowledge; 2. Gather simulated monitoring data from the operating model; 3. Analyze the monitoring data using an estimation model and potentially use informed priors generated through Bayesian updating, then predict population dynamics; 4. Based on the estimated model predictions, update the allocation of management effort, which subsequently influences the realistic population dynamics of the species in the operating model. The details in italics are specific to this study. Figure was adapted from Siple et al. (2021).

The MSE process involves four main steps (Siple et al. 2021, Figure 2). First, under multiple alternative management strategies, we simulate realistic system dynamics and management using distributions of parameter values representing the current state of knowledge in a model denoted as the “operating model.” Second, we simulate the collection of monitoring data using the operating model. Third, we analyze the monitoring data using an “estimation model” (e.g., an occupancy model; MacKenzie et al. 2003), potentially using informed parameter priors generated through Bayesian updating, and predict system dynamics. Fourth, based on model predictions, we revise the management decision (i.e., the spatial allocation of monitoring and removal effort), which subsequently influences system dynamics simulated in the operating model (Figure 2). Hence, this process involves the use of two models. One model (the operating model) simulates “reality,” or what is truly happening in the simulated system of interest, while the second model (the estimation model) simulates what a manager would have available to them to inform their decision at a given point in time.

### 2.3 Management and Monitoring Alternatives

The management and monitoring alternatives we developed and simulated were a function of four key elements: (1) spatial priority, referring to the locations of segments that were prioritized for monitoring and management; (2) target detection and eradication probabilities per segment; (3) investment, referring to the hours of labor per week deployed to monitor and manage flowering rush; and (4) types of monitoring data gathered (Table 1). We describe each of these in order below.

**FIGURE 3:**
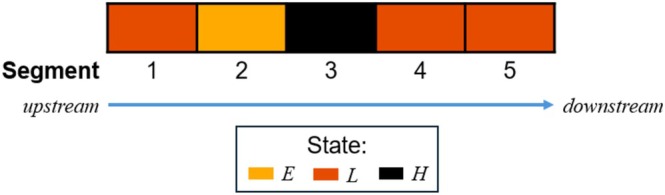
Illustration of the three spatial priority actions using a simplified river of five total segments. The arrows depict the flow of the river from upstream to downstream. Prior to management, the invasion state of each segment is estimated: Empty (*E*), low severity (*L*), or high severity (*H*). The colors depict a possible outcome of this estimation process for the 5‐segment example. If the *Linear* spatial priority action was selected, the order of segment visitation would be: 1, 2, 3, 4, 5. If the *high invasion* spatial priority action was selected, the order of segment visitation would be: 3, 4, 5, 1, 2. If the e*picenter* spatial priority action was selected, the order of segment visitation would be: 3, 4, 5, 2, 1.

First, spatial priority describes the order in which segments were visited in the simulation during the 4‐week removal period each year. We developed three prioritization approaches: *Linear*, *High invasion*, and *Epicenter*, inspired by conversations with managers in the study area (Figure 3). The *Linear* approach prioritized segments according to location, starting from the most upstream segment (Figure 3). Under this approach, information about the invasion state of segments did not inform management—segment visits were simulated in a fixed order. In the two other approaches, however, information about the invasion state did inform spatial prioritization. In the context of a multistate occupancy model (see Section 2.4) we used monitoring data to estimate the invasion severity state of segments, either unoccupied (state = 0), low severity (state = 1), or high severity (state = 2). Specifically, we used the posterior mean of the invasion state parameter as a measure of invasion severity. Under *High invasion*, segments were prioritized by estimated severity state and visited in order according to decreasing severity state, starting from the most upstream river segment (Figure 3). Under *Epicenter*, we identified the single segment with the most severe estimated invasion state, and segments were visited outwards from this “epicenter” in the direction of the highest adjacent invasion state, then were visited outwards from the “epicenter” in the other direction (Figure 3). In each of the alternatives, *Linear* prioritization was applied during the first 3 years of management to allow for learning about the relationship between effort and probabilities of detection and eradication. After this, the time allocated to searching or managing in any segment was fixed under each alternative. In addition, in all three prioritization approaches, parameters (e.g., invasion growth and spread rates) were updated throughout the simulation to improve predictions of system function, allowing for improved prediction of system state. In the *High invasion* and *Epicenter* prioritization actions, the segment prioritization lists were updated based on these predictions.

Second, target detection and eradication probabilities refer to target probabilities at visited segments. We assumed that detection and eradication probabilities were a function of the number of hours spent searching for and removing flowering rush, respectively. As for many invasive species, there is substantial uncertainty regarding the amount of time that it takes to achieve a given detection or eradication probability for flowering rush. Therefore, to meet targets, we assumed that managers would first need to learn about the relationship between the number of hours spent searching in a segment and the resulting detection probability. They would also need to learn about the relationship between the number of hours spent removing the plant in a segment and the resulting eradication probability. In both cases, it is reasonable to assume that these probabilities increase with increasing effort, in some likely nonlinear fashion. To provide for this learning, during the first 3 years of simulated management, when *Linear* prioritization was conducted, we assigned random search and removal hours to each visited segment from a Uniform (0.1, 10) distribution, covering a wide, yet reasonable, maximum search effort range, which allowed us to estimate the relationship between effort and probabilities of detection and eradication. Then, after 3 years of management, we found the number of search hours and removal hours that allowed us to achieve each of the six sets of target detection, *p*, and eradication, ϵ, probabilities (Table 1), averaged across invasion severity, and these search and effort hours were then fixed for the relevant management alternative. We assumed goal detection probabilities were either 0.25, 0.5, or 0.75 and goal eradication probabilities were 0.5 or 0.75. Due to the difficulty of detecting and eradicating flowering rush (Columbia Basin CWMA 2019), we did not evaluate probabilities beyond 0.75.

Third, investment refers to different budget levels, expressed as the maximum labor hours (h) available each week: 20, 40, or 60 h, referred to as low, medium, and high investment. The budget was expended through hours spent searching for and removing flowering rush, and hours spent traveling between segments (assuming boats with agency workers traveled 10 knots or 18.5 km/h). Prior to the first week of removal each year, the spatial priority list was developed, segments were visited in that order, and visitation ended each week when all the segments were visited or when the budget was exhausted, whichever occurred first. If the budget was exhausted, the locations for the start of the next week would begin at the next segment on the spatial priority list.

Fourth, the types of monitoring data gathered included either (1) only the data collected during detection and management (i.e., multistate data describing observed invasion status) or (2) the data collected during detection and management as well as additional community science data. We assumed that in pairs, community scientists visited eight randomly selected segments each week for 1 h per segment and collected single‐state detection/nondetection data for those segments. We also assumed that the time volunteers spent collecting data did not reduce the available agency budget. We aimed to determine whether the addition of opportunistic community science data would help achieve the management objective by contributing to the ability to predict the invasion state of segments. This was tested through the integration of community science data for alternatives that included *High invasion* and *Epicenter* prioritization.

In total, we evaluated 54 management alternatives in which the agency was collecting all the monitoring data, including three spatial prioritizations, six sets of target probabilities for detection and eradication, and three investment levels. We evaluated six additional alternatives with the addition of community science data, including two spatial prioritizations (*High invasion* and *Epicenter*), one set of target probabilities (the set that led to the best outcomes for the respective prioritization and investment level in the absence of community science data, and three investment levels). We also simulated a *No removals* alternative where no monitoring or removal of flowering rush occurred (Table 1).

### 2.4 Modeling Framework

We developed a dynamic multistate occupancy model for this study (MacKenzie et al. 2009). Dynamic occupancy models have been used to model the distribution and dynamics of invasive plants and other invasive species (Bled et al. 2011; Pepin et al. 2019; Middleton and Vining 2022). We used this model approach because we aimed to estimate different invasion severity states (MacKenzie et al. 2017), rather than just invaded/not invaded, and we wanted to incorporate realistic dynamics of invasion (i.e., site colonization). Both the operating and estimation models were dynamic multistate models; the operating model simulated the true latent population process using the true latent parameter values, while the estimation model relied on the simulated monitoring data to estimate occupancy parameters. An extension of the estimation model was developed to integrate community science data.

In the model, each river segment was in one of three invasion severity states at each point in time: no invasion, referred to as empty (denoted as *E* = 0), low severity (*L* = 1) in which the plant occupies less than 10% of available habitat in the segment, and high severity (*H* = 2) in which the plant occupies more than 10% of available habitat in a segment. Together, invaded states *L* and *H* are sometimes referred to as the collective occupied states, *S*. We indexed segments with *i* (1, 2, …, *I* = 40), removal weeks within a year with *w* (1, 2, …, *W* = 5), and years with *t* (1, 2, …, *T* = 10). The timing of events is important due to the seasonal growth of flowering rush, and because monitoring and removal only occur for 4 weeks a year. Therefore, our modeling framework was divided into two periods. The first period, the “data period,” covered the 4 weeks during which monitoring and removal were simulated. The second period, the “nondata period,” covered the remaining weeks each year, when monitoring and removal were not occurring. In Table 2 we provide a summary of parameter values used in both the state transition process and the data observation process for each time period in the model.

**TABLE 2:** Description of parameters in our simulation and estimation models for a management strategy evaluation for invasive flowering rush.

Parameter	Description	Model
$z_{i,w,t}$	Invasion state	State transition (both periods)
$\Phi_{i,w,t}^{\left[D\right]}$	Transition probability matrix	State transition (data period)
$\epsilon_{i,w,t}^{\left[S\right]}$	Eradication probability for state *S*	State transition (data period)
$\beta_{0}^{\left[\epsilon ,S\right]}$	Logit‐scale eradication probability for state *S* in the absence of management	State transition (data period)
$\beta_{1}^{\left[\epsilon ,S\right]}$	Logit‐scale effect of removal effort on eradication probability	State transition (data period)
$\varphi_{i,w,t}^{\left[H\right]}$	Probability of staying in invaded state *H*	State transition (data period)
$\beta_{0}^{\left[\varphi ,H\right]}$	Logit‐scale probability of staying in state *H* in the absence of management	State transition (data period)
$\beta_{1}^{\left[\varphi ,H\right]}$	Logit‐scale effect of removal effort on the probability of staying in state *H*	State transition (data period)
$\alpha_{i,t}$	Vector of the probability of being in state *E*, *L* and *H* on week 5 for year *t*	State transition (data period)
$y_{i,w,t}^{\left[O\right]}$	Observation data for observation type O	Observation (both data types)
$P_{i,w,t}^{\left[O\right]}$	Detection probability matrix	Observation (both data types)
$p_{i,w,t}^{\left[S,O\right]}$	Detection probability under state *S* for data type *O*	Observation (both data types)
$\beta_{0}^{\left[p,S,O\right]}$	Logit‐scale detection probability for state *S* and data type *O* with no search effort	Observation (both data types)
$\beta_{1}^{\left[p,S,O\right]}$	Logit‐scale effect of log(search effort) on detecting state *S* for data type *O*	Observation (both data types)
$M_{i,w,t}^{\left[O\right]}$	Search effort hours for data type *O*	Observation (both data types)
δ	Probability of correctly observing state *H*	Observation (agency model)
$\Phi_{i,t}^{\left[B\right]}$	Transition probability matrix	State transition (nondata period)
$\gamma_{i,t}$	Invasion probability	State transition (nondata period)
$\beta_{0}^{\left[\gamma \right]}$	Logit‐scale invasion probability in the absence of management	State transition (nondata period)
$\beta_{1}^{\left[\gamma \right]}$	Logit‐scale effect of habitat on invasion probability	State transition (nondata period)
$h_{i}$	Segment‐specific habitat covariate	State transition (nondata period)
$\beta_{2}^{\left[\gamma \right]}$	Logit‐scale effect of adjacent invasion state on invasion probability	State transition (nondata period)
$N_{i,t}$	The state of neighboring segments	State transition (nondata period)
$d^{\left[S\right]}$	Eradication probability for state *S* from the end of one data period to the start of the next data period (between data periods)	State transition (nondata period)
$r^{\left[S\right]}$	Probability of remaining in state *S* between data periods	State transition (nondata period)
g	Probability of being in state *H* if invaded between data periods	State transition (nondata period)

*Note:* Simulation model parameters used in a state transition model apply to the data period (the weeks with active management and monitoring), the nondata period (the period between the end of one data period and the start of the next), or both. Parameters appearing in the simulation model also appear in the estimation model, an occupancy model. The occupancy model includes detection parameters (associated with either agency or community science observations). Parameter indices include *i* for segments, *w* for weeks, and *t* for year. State *E* refers to the empty state. Invaded state *S* refers to low severity (*L*) or high severity (*H*). For the observation data, *O* is replaced by *A* to represent agency or *C* to represent community science observations.

#### 2.4.1 Data Period State Transition Model

During the data period, starting on August 1, and given an estimate of the state of the river for week 1 (see Section 2.5 for description of initial invasion states for year 1), we simulate observations and removal of flowering rush by the agency and, depending on the alternative, observations by the community scientist volunteers. Then, at the end of week 1, state transitions occur to advance to the start of week 2. For three more weeks, we generate data, simulate removals and state transitions, and model state transitions to the start of week 5, during which no removals occur. Throughout the data period, we assume no population change other than through removals and natural mortality because, by late summer, the biomass of flowering rush has reached its maximum value for the year and remains dormant until it senesces during winter (Columbia Basin CWMA 2019). Thus, a segment with a true state of *E =* 0 in week 1 will remain in *E =* 0 throughout the data period, though segments in a true state of *L* = 1 or *H* = 2 can change state due to management or mortality.

We modeled the state transitions between weeks during the data period as:

(1) $$z_{i,w+1,t}|z_{i,w,t}\sim \text{Categorical}\left(\Phi_{i,w,t}^{\left[D\right]}\right),$$

where $z_{i,w,t}$ is the invasion state at segment *i*, in week *w* (*w* = 2–5), during year *t* (*t* = 1–10), which is either *E*, *L*, or *H* (with numerical values of 0, 1, or 2). The transition probability matrix during the data period, $\Phi_{i,w,t}^{\left[D\right]}$, describes the probability of moving from $z_{i,w,t}$ in week w to state $z_{i,w+1,t}$ in week *w* + 1, where *D* denotes the data period. Equation (2) provides the transition probability matrix, $\Phi_{i,w,t}^{\left[D\right]}$, with the state during week *w* in the rows and the state during week *w* + 1 in the columns:

(2) $$\Phi_{i,w,t}^{\left[D\right]}=\begin{array}{cc} \begin{array}{c} \begin{array}{c} E\\ L\\ H \end{array} \end{array} & \begin{array}{c} E\\ \left[\begin{array}{c} 1\\ \epsilon_{i,w,t}^{\left[L\right]}\\ \epsilon_{i,w,t}^{\left[H\right]} \end{array}\right. \end{array} \end{array}\;\begin{array}{cc} \begin{array}{c} L\\ \begin{array}{c} 0\\ 1-\epsilon_{i,w,t}^{\left[L\right]}\\ \left(1-\epsilon_{i,w,t}^{\left[H\right]}\right)\left(1-\varphi_{i,w,t}^{\left[H\right]}\right) \end{array} \end{array} & \begin{array}{c} H\\ \left.\begin{array}{c} 0\\ 0\\ \left(1-\epsilon_{i,w,t}^{\left[H\right]}\right)\varphi_{i,w,t}^{\left[H\right]} \end{array}\right] \end{array} \end{array}.$$

We modeled eradication probability, or the transition probability from the invaded state *S* (*L* or *H*) to the empty state *E* as:

(3) $$\text{logit}\left(\epsilon_{i,w,t}^{\left[S\right]}\right)=\beta_{0}^{\left[\epsilon ,S\right]}+\beta_{1}^{\left[\epsilon ,S\right]}X_{i,w,t},$$

where $\beta_{0}^{\left[\epsilon ,S\right]}$ is the logit‐scale state‐specific eradication probability when no removal effort is conducted (i.e., natural mortality), $\beta_{1}^{\left[\epsilon ,S\right]}$ is the state‐specific effect of per‐hour removal effort on eradication probability (such that $\beta_{1}^{\left[\epsilon ,S\right]}$ is expected to be positive), and $X_{i,w,t}$ is the removal effort (hours) expended at segment *i* during week *w* and year *t*. We modeled the probability of remaining in state *H* as:

(4) $$\text{logit}\left(\varphi_{i,w,t}^{\left[H\right]}\right)=\beta_{0}^{\left[\varphi ,H\right]}+\beta_{1}^{\left[\varphi ,H\right]}X_{i,w,t}$$

where $\beta_{0}^{\left[\varphi ,H\right]}$ is the logit‐scale probability of staying in state *H* (i.e., not transitioning to *L*) given no removal effort, and $\beta_{1}^{\left[\varphi ,H\right]}$ is the effect of per‐hour removal effort on this probability (such that $\beta_{1}^{\left[\varphi ,H\right]}$ is expected to be negative).

The transition process in Equation (1) estimates invasion severity states for *w =* 2–5. In the estimation model, for week 1, year 1, we let $z_{i,w=1,t=1}\sim \text{Categorical}\left(\text{Dirichlet}\left(1,1,1\right)\right)$ which places an equal weight on being in each of the three states. For future years, t+1, we estimate $z_{i,w=1,t+1}$ given $z_{i,w=5,t}$ (see Section 2.4.3 details below, Equation 9) and in the estimation model we set $z_{i,w=5,t}\sim \text{Categorical}\left(\alpha_{i,t}\right)$ with $\alpha_{i,t}$ drawn from a Dirichlet distribution with parameters describing the estimated probability of being in each of the three states on week 5 during year *t* (i.e., an output of the estimation model calculation of $z_{i,w=5,t}$).

#### 2.4.2 Observation Models

Next, we describe the observation process (i.e., imperfect detection of invasion states) for both agency and community science data streams during the data period. We use the notation *O* to indicate the observation data, where *O* is replaced by *A* to represent agency data and *O* is replaced by *C* to represent community science data. Both types of data are drawn from a categorical distribution, conditional on the true occupancy state:

(5) $$y_{i,w,t}^{\left[O\right]}|z_{i,w,t}\sim \text{Categorical}\left(P_{i,w,t}^{\left[O\right]}\right).$$

The agency multistate detection/nondetection data, denoted as $y_{i,w,t}^{\left[A\right]}$, can take values of 0 for nondetection, 1 for observing the state as *L*, and 2 for observing the state as *H*. Hence, the detection probability matrix for agency data, $P_{i,w,t}^{\left[A\right]}$ with the columns representing observations and the rows representing the true states, is expressed as:

(6) $$P_{i,w,t}^{\left[A\right]}=\begin{array}{cc} \begin{array}{cc} \begin{array}{c} \begin{array}{c} E\\ L\\ H \end{array} \end{array} & \begin{array}{c} 0\\ \left[\begin{array}{c} 1\\ 1-p_{i,w,t}^{\left[L,A\right]}\\ 1-p_{i,w,t}^{\left[H,A\right]} \end{array}\right. \end{array} \end{array} & \begin{array}{cc} \begin{array}{c} 1\\ \begin{array}{c} 0\\ p_{i,w,t}^{\left[L,A\right]}\\ p_{i,w,t}^{\left[H,A\right]}\left(1-\delta \right) \end{array} \end{array} & \begin{array}{c} 2\\ \left.\begin{array}{c} 0\\ 0\\ p_{i,w,t}^{\left[H,A\right]}\delta \end{array}\right] \end{array} \end{array}. \end{array}$$

In contrast, state detection/nondetection community science data, denoted as $y_{i,w,t}^{\left[C\right]}$, can take only values of 0 for nondetection and 1 for detection, and the detection probability matrix, $P_{i,w,t}^{C}$, is:

(7) $$P_{i,w,t}^{C}=\begin{array}{cc} \begin{array}{cc} \begin{array}{c} \begin{array}{c} E\\ L\\ H \end{array} \end{array} & \begin{array}{c} 0\\ \left[\begin{array}{c} 1\\ 1-p_{i,w,t}^{\left[L,C\right]}\\ 1-p_{i,w,t}^{\left[H,C\right]} \end{array}\right. \end{array} \end{array} & \begin{array}{c} 1\\ \left.\begin{array}{c} 0\\ p_{i,w,t}^{\left[L,C\right]}\\ p_{i,w,t}^{\left[H,C\right]} \end{array}\right] \end{array}. \end{array}$$

We modeled probability for each observation type (*O = A* or *C*) under invaded state *S* as:

(8) $$\text{logit}\left(p_{i,w,t}^{\left[S,O\right]}\right)=\beta_{0}^{\left[p,S,O\right]}+\beta_{1}^{\left[p,S,O\right]}\log\left(M_{i,w,t}^{\left[O\right]}\right)$$

and we assumed the agency and community scientists had different detection probabilities, so the intercept, slope, and covariate values (Equation 8) were different between agency (*O* = *A*) and community science (*O* = *C*) observations (Table S1). We let $\beta_{0}^{\left[p,S,O\right]}$ be the state‐specific logit‐scale detection probability when search effort is zero. We assumed a logarithmic relationship between hours spent monitoring and detection probability, such that detection probability is forced to 0 when effort is 0. Hence, $\beta_{1}^{\left[p,S,O\right]}$ is the state‐specific effect of log(search hours), that is, $\log\left(M_{i,w,t}^{\left[O\right]}\right)$, on detection probability for observation type *O*. However, for community science data, we assumed $M_{i,w,t}^{\left[C\right]}=1\;$ h in all cases, and the detection Equation (8) simplifies to $\text{logit}\left(p_{i,w,t}^{\left[S,C\right]}\right)=\beta_{0}^{\left[p,S,C\right]}$.

Since we simulate collection of multistate detection data by the agency, we define δ in row 3 of the detection probability matrix, $P_{i,w,t}^{\left[A\right]}$ (Equation 6), as the probability of observing state *H* given the species has been detected and the true state is *H*, while 1−δ is the probability of observing state *L* given the species has been detected and the true state is *H*; in other words, we allow for state uncertainty in the observation process (MacKenzie et al. 2009).

#### 2.4.3 Nondata Period State Transition Model

Next, we describe the nondata period during which no data collection or removals occurred (between the start of week 5 and the beginning of week 1 in the next year). During this period, state transitions occurred as a function of population growth during the seasonal growth period in the spring and early summer. We modeled the transition of the invasion state from the end of data collection in the previous year (*w* = 5, *t*−1) to the initiation of management and data collection in the current year (*w* = 1, *t*) as:

(9) $$z_{i,1,t}|z_{i,5,t-1}\sim \text{Categorical}\left(\Phi_{i,t}^{\left[B\right]}\right),$$

where $\Phi_{i,t}^{\left[B\right]}$ is the transition probability matrix during the nondata period, denoted as *B* (i.e., different from Equation 2). Note the absence of the *w* subscript, as there are no weekly time steps during this period:

(10) $$\Phi_{i,t}^{\left[B\right]}=\begin{array}{cc} \begin{array}{cc} \begin{array}{c} \begin{array}{c} E\\ L\\ H \end{array} \end{array} & \begin{array}{c} E\\ \left[\begin{array}{c} 1-\gamma_{i,t}\\ d^{\left[L\right]}\\ d^{\left[H\right]} \end{array}\right. \end{array} \end{array} & \begin{array}{cc} \begin{array}{c} L\\ \begin{array}{c} \gamma_{i,t}\left(1-g\right)\\ \left(1-d^{\left[L\right]}\right)r^{\left[L\right]}\\ \left(1-d^{\left[H\right]}\right)\left(1-r^{\left[H\right]}\right) \end{array} \end{array} & \begin{array}{c} H\\ \left.\begin{array}{c} \gamma_{i,t}g\\ \left(1-d^{\left[L\right]}\right)\left(1-r^{\left[L\right]}\right)\\ \left(1-d^{\left[H\right]}\right)r^{\left[H\right]} \end{array}\right] \end{array} \end{array}. \end{array}$$

Invasion probability, the probability of segment *i* transitioning from state *E* to an invaded state *S*, is modeled as:

(11) $$\text{logit}\left(\gamma_{i,t}\right)=\beta_{0}^{\left[\gamma \right]}+\beta_{1}^{\left[\gamma \right]}h_{i}+\beta_{2}^{\left[\gamma \right]}N_{i,t-1}$$

where $\beta_{0}^{\left[\gamma \right]}$ is the logit‐scale intercept for invasion probability, $\beta_{1}^{\left[\gamma \right]}$ is the logit‐scale effect of segment habitat ($h_{i}$) on invasion probability, and $\beta_{2}^{\left[\gamma \right]}$ is the logit‐scale effect of the state of neighboring segments at week 5 in the previous year on invasion probability. The state of neighboring segments was expressed as:

(12) $$N_{i,t-1}=\frac{\sum_{l\in n\left(i\right)}z_{l,5,t-1}}{|n\left(i\right)|},$$

where *n*(*i*) is the set of segments neighboring segment *i*, *l* is an element of that set, and |*n*(*i*)| is the size of that set. For segments 1 and 40 (i.e., the ‘edge’ of the study area), |*n*(*i*)| = 1, and the numerator of Equation (12) can take values of 0, 1, or 2. For all other segments, |*n*(*i*)| = 2 (i.e., all segments except the ‘edges’ have two neighboring segments) and the numerator of Equation (12) takes integer values between 0 and 4. At the start of simulations, $h_{i}$, representing whether a segment has habitat characteristics that promote establishment of flowering rush, with a value of 1 indicating ideal conditions, was drawn from a Uniform(0, 1) distribution and was held constant throughout all simulations. Parameter *g* in the transition matrix (Equation 10) is the probability of transitioning to state *H* given that colonization occurred. The eradication probability for invaded state *S* during this period is $d^{\left[S\right]}$ and the probability of remaining in invaded state *S*, that is, not transitioning to *L* for a state in *H* or not transitioning to *H* for a state in *L*, is $r^{\left[S\right]}$. Since there was no removal effort during this period, the eradication probability is equivalent to natural local extinction.

At the end of the nondata period, we have an estimate of the state at each segment in week 1 of the next management year (Equation 9). Using this information, we generate our segment prioritization list (Table 1), and then we conduct the data collection and removal process for the following 4 weeks (Equations 1 and 5). Then, we again project the state transitions during the nondata period (Equation 9) and so on, continuing this process for 10 years (Figure 2).

For the estimation portion of our MSE (Figure 2), the occupancy model likelihood for alternatives with only agency data were $L^{\left[A\right]}\left(\Phi_{i,w,t}^{\left[D\right]},\Phi_{i,t}^{\left[B\right]},P_{i,w,t}^{\left[A\right]}|y_{i,w,t}^{\left[A\right]}\right)$. For alternatives with both agency and community science data, we used an integrated model with a joint‐likelihood of $L^{\left[A\right]}\left(\Phi_{i,w,t}^{\left[D\right]},\Phi_{i,t}^{\left[B\right]},P_{i,w,t}^{\left[A\right]}|y_{i,w+1,t}^{\left[A\right]}\right)\times L^{\left[C\right]}\left(\Phi_{i,w,t}^{\left[D\right]}\Phi_{i,t}^{\left[B\right]},P_{i,w,t}^{\left[C\right]}|y_{i,w,t}^{\left[C\right]}\right)$.

### 2.5 Simulation Procedure

Simulations were coded in R (version 4.3.1; R Core Team 2023), with an embedded MCMC estimation process for fitting the occupancy model to simulated monitoring data, coded in JAGS (version 4.3.2; Plummer 2003). In the MCMC process, we used 3 chains with 20,000 iterations and a burn‐in value of 2000, for a posterior distribution of 54,000 samples. We evaluated model convergence by confirming that model parameters had a Gelman‐Rubin diagnostic value < 1.1 across all simulations and years. We also evaluated convergence by visually inspecting a subset of the trace plots and density plots. Due to the need for an initial estimate of the transition probabilities during the nondata weeks period (i.e., parameters in $\Phi_{i,t}^{\left[B\right]}$) we ran our estimation model with 2 years of monitoring data (Figure S1) before updating management. Then for subsequent years, we ran the estimation model for just a year at a time and updated our segment prioritization list based on estimated results.

Published literature supported the specification of the statistical distributions for the parameters within the operating model. Table S1 depicts the statistical distributions, and we drew each parameter value from the respective distribution 200 times to create 200 unique parameter sets. Each alternative was run under each of the 200 parameter sets. These values represented “truth,” and with each of the 200 parameter sets, the initial priors for every parameter in the first run of the estimation model (i.e., year 1 and year 2) were drawn from the distribution depicted in Table S1. After the first run of the estimation model (i.e., starting with year 3), all parameter priors were updated via Bayesian updating (Applestein et al. 2022).

**FIGURE 4:**
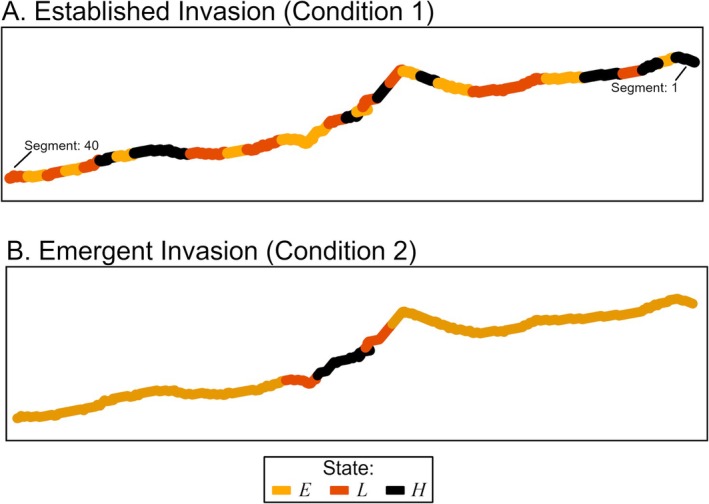
Two initial invasion conditions of flowering rush simulated across the 40‐segment state space for the case study, including (A) initial invasion states by segment under condition 1, an established invasion, and (B) initial invasion states by segment under condition 2, an emerging invasion. The colors depict the initial true invasion state at each segment with orange depicting *E*, empty, red depicting *L*, low severity, and black depicting *H*, high severity. Segment 1 is the most upstream segment in the region and Segment 40 is the most downstream segment.

In addition, since the initial distribution of an invasive species can impact the rate of spread and the effectiveness of spatial allocation strategies, and thereby management outcomes (Carter et al. 2018), we tested all alternatives under two different invasion conditions in place at the beginning of the management simulation. Condition 1, “established invasion,” represented an invasion with flowering rush distributed across many segments, and condition 2, “emerging invasion,” represented an invasion with the species distributed in only a few segments (Figure 4). In condition 1, initially 35% of the river was in state *E*, 37.5% was in state *L*, and 27.5% was in state *H*. In condition 2, initially 80% of the river was in state *E*, 10% was in state *L*, and 10% was in state *H*. These initial values describe the true, operating model starting states.

### 2.6 Evaluation of Alternatives

We evaluated the alternatives in terms of our management objective after 10 years of management. We calculated the performance on our objective, “suppression” (i.e., to minimize overall invasion), for each simulation as the average state of the system at week 5 after 10 years of management, $\sum_{i=1}^{I=40}\frac{z_{i,5,10}}{I}$. We found the expected value and the maximum value of this quantity across parameter sets for each alternative. We then identified optimal alternatives based on two decision criteria: maximum expected value and mini–max. The alternative that maximizes expected value is the alternative with the best average outcome for an objective across simulations and is commonly used for risk‐neutral decision makers. The mini–max criterion selects the alternative that has the “best worst outcome” for the objective, which accounts for risk‐aversion (Runge and Converse 2020). That is, since our management objective was to minimize invasion, we identified the maximum invasion outcome (i.e., performance of alternatives in terms of the suppression objective, as described above) for each alternative across simulations, and then we identified the alternative with the minimum of these maximum values as the alternative that performed best on the mini–max criterion.

Finally, we were interested in the relationship between management performance and estimation performance and so we examined estimation performance under each alternative for various parameter values by calculating relative bias and root mean square error (RMSE) across several parameters. We evaluated what we refer to as “state relative bias” and “state RMSE” which are the bias and RMSE, respectively, in the estimated state of each segment at the start of week 5 in each year. We also calculated “*p* relative bias” and “*p* RMSE” which are the average bias and RMSE across the estimated agency detection parameters, $\beta_{0}^{\left[p,L.A\right]}$, $\beta_{1}^{\left[p,L.A\right]}$, $\beta_{1}^{\left[p,L.A\right]}$, $\beta_{1}^{\left[p,H.A\right]}$; and “ϵ relative bias” and “ϵ RMSE,” the average bias and RMSE across the estimated eradication parameters $\beta_{0}^{\left[\epsilon ,L\right]}$, $\beta_{1}^{\left[\epsilon ,L\right]}$, $\beta_{0}^{\left[\epsilon ,H\right]}$, $\beta_{1}^{\left[\epsilon ,H\right]}$.

## 3 Results

**FIGURE 5:**
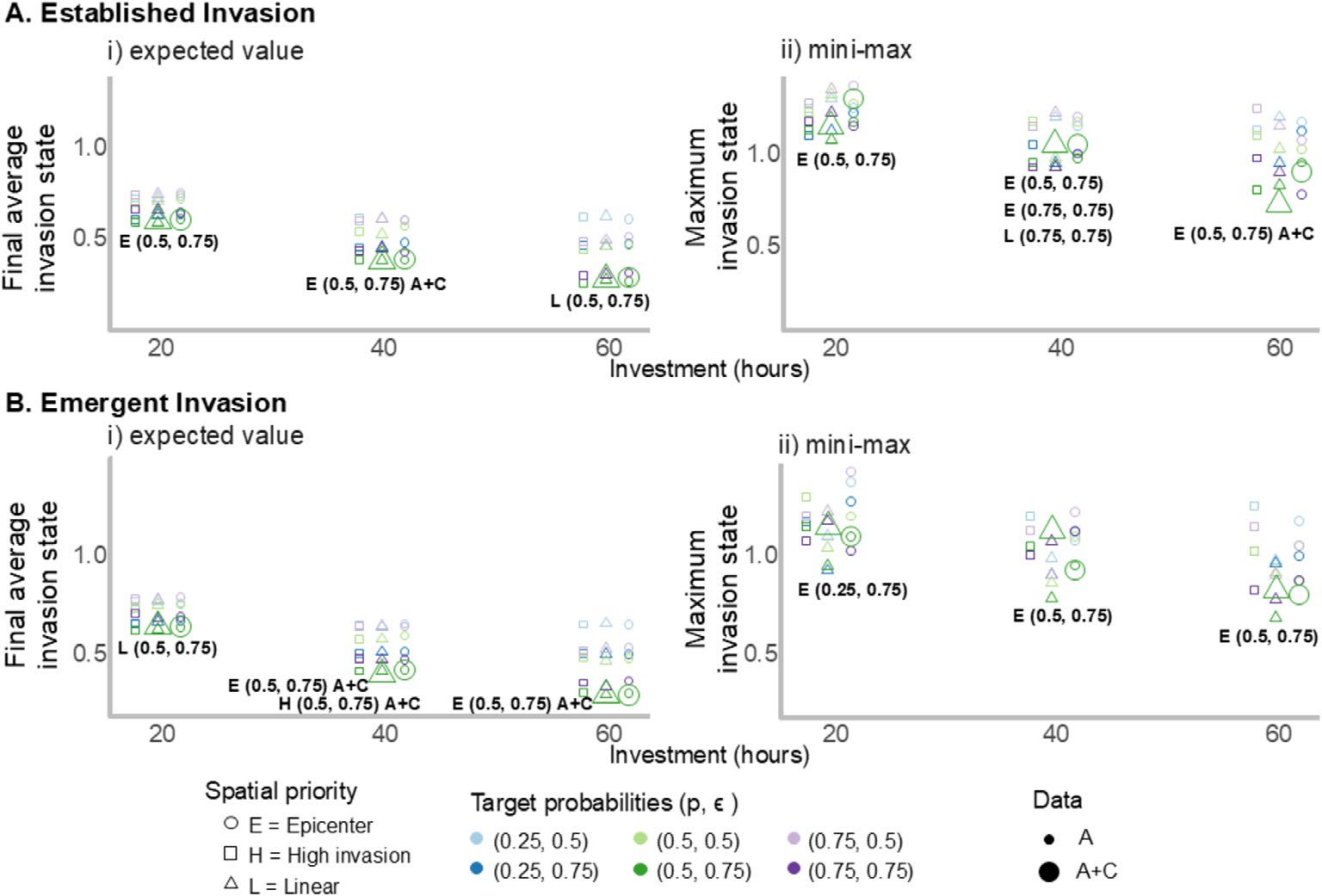
Performance of each simulated alternative in meeting the management objective to minimize the final average invasion state of flowering rush in the Columbia River study area, displayed across the three investment levels (20, 40, and 60 h of effort per week). A final average invasion state of 0 indicates eradication while a final average invasion state of 2 indicates that all segments are in the high invasion state. In box A, outcomes are displayed for an established invasion, and in box B, outcomes are displayed for an emerging invasion. In plots A and B, subplot (i) displays the expected‐value outcome for each alternative across simulations for each investment level; the bolded text displays the alternative that performed best in terms of expected value. The expected‐value criterion is used for risk‐neutral decision makers. In plots A and B, subplot (ii) displays the maximum outcome for each alternative across simulations and for each investment level, the bolded text displays the alternative that performed best in terms of minimizing the maximum potential invasion outcome, or the “mini–max” criterion. This criterion is used for risk‐averse decision makers. The alternatives are a function of spatial priority (shown with the shapes), target detection and eradication probabilities (shown with colors), and the data used in estimation (shown with the size of the points) where “A” indicates just agency were collected, and “A + C” indicates agency and community science data were collected. In the bolded text, we indicate whether the top alternative included community science data with “A + C.”

For an established invasion, *Epicenter* prioritization with a target detection probability (*p*) of 0.5 and eradication probability (ϵ) of 0.75 was the optimal alternative across most investment levels for both risk‐neutral (i.e., expective value choice) and risk‐averse (i.e., mini‐max choice) decision makers (Figure 5A, Table S2, Figure S2). However, *Linear* prioritization with target probabilities of 0.5 and 0.75, for *p* and ϵ, respectively, was preferred for a risk‐neutral decision maker with a high investment (Figure 5A, Table S2). For a risk‐averse decision maker with a moderate investment, *Linear* prioritization with target probabilities of 0.75 and 0.75 and *Epicenter* prioritization with target probabilities of 0.5 and 0.75, or 0.75 and 0.75, for *p* and ϵ, respectively, were all equally preferred (Figure 5Aii, Table S2). We found that integrating community science data was only useful for a risk‐neutral decision maker with a moderate investment and a risk‐averse decision maker with a high investment (Figure 5A, Table 3).

**TABLE 3:** Results under both invasion conditions for a subset of alternatives with the addition of community science data.

Alternative				Objective		Model performance	
				Suppression aim to minimize		Invasion relative bias aim for zero	
Investment (hours/week)	Spatial priority	Target probabilities (*p*, ϵ)	Data	Mean outcome	Max outcome	Mean outcome	Max outcome
Established invasion (Condition 1)							
None (0)	None	(0, 0)	None	0.934	1.300	NA	NA
Low (20)	*High invasion*	(0.5, 0.75)	A	0.638	1.180	0.166	1.75
		(0.5, 0.75)	A + C	0.636	1.300	0.216	1.82
	*Epicenter*	(0.5, 0.75)	A	0.617	1.080	0.175	1.74
		(0.5, 0.75)	A + C	0.630	1.15	0.205	1.83
	*Linear*	(0.5, 0.75)	A	0.618	1.120	0.167	1.75
Moderate (40)	*High invasion*	(0.5, 0.75)	A	0.416	0.975	0.129	1.77
		(0.5, 0.75)	A + C	0.417	1.050	0.148	1.83
	*Epicenter*	(0.5, 0.75)	A	0.416	0.950	0.130	1.78
		(0.5, 0.75)	A + C	0.409	1.050	0.149	1.81
	*Linear*	(0.5, 0.75)	A	0.416	0.950	0.131	1.78
High (60)	*High invasion*	(0.5, 0.75)	A	0.299	0.950	0.121	1.78
		(0.5, 0.75)	A + C	0.320	0.900	0.139	1.81
	*Epicenter*	(0.5, 0.75)	A	0.309	0.825	0.117	1.79
		(0.5, 0.75)	A + C	0.308	0.725	0.119	1.85
	*Linear*	(0.5, 0.75)	A	0.290	0.800	0.115	1.76
Emergent invasion (Condition 2)							
None (0)	None	(0, 0)	None	0.941	1.375	NA	NA
Low (20)	*High invasion*	(0.5, 0.75)	A	0.631	1.100	0.178	1.68
		(0.5, 0.75)	A + C	0.635	1.100	0.199	1.77
	*Epicenter*	(0.5, 0.75)	A	0.619	0.950	0.166	1.66
		(0.5, 0.75)	A + C	0.634	1.150	0.202	1.79
	*Linear*	(0.5, 0.75)	A	0.612	1.150	0.175	1.71
Moderate (40)	*High invasion*	(0.5, 0.75)	A	0.415	0.950	0.132	1.75
		(0.5, 0.75)	A + C	0.415	0.925	0.150	1.79
	*Epicenter*	(0.5, 0.75)	A	0.407	0.783	0.135	1.76
		(0.5, 0.75)	A + C	0.392	1.120	0.152	1.81
	*Linear*	(0.5, 0.75)	A	0.407	1.050	0.134	1.76
High (60)	*High invasion*	(0.5, 0.75)	A	0.297	0.875	0.103	1.81
		(0.5, 0.75)	A + C	0.285	0.800	0.119	1.84
	*Epicenter*	(0.5, 0.75)	A	0.287	0.683	0.108	1.78
		(0.5, 0.75)	A + C	0.285	0.825	0.122	1.83
	*Linear*	(0.5, 0.75)	A	0.296	0.825	0.106	1.76

*Note:* We display results first for an established invasion (condition 1), followed by results for an emergent initial invasion (condition 2). We show the performance of integration of community science data (noted by Data A + C) compared to no community science data (A) and further compare outcomes against *Linear* (0.5, 0.75). For each investment level, the best‐performing alternative and associated outcome are colored in green, and the worst is colored in gray. The outcomes identified from the model simulations included the mean and maximum outcome in the suppression objective (final average invasion state) across simulations. A model performance metric was identified for each alternative. We display the relative bias of invasion severity (noted as Invasion Relative Bias). We show the mean and maximum relative bias calculations across simulations with high values indicating an overestimation of invasion severity.

Under an emerging invasion condition, integrating community science data improved management outcomes for a risk‐neutral decision maker with moderate or high investment (Figure 5B, Table S2, Figure S3). Specifically, the *Epicenter* prioritization with target detection and eradication probabilities of 0.5 and 0.75, respectively, with the addition of community science data, was preferred for a risk‐neutral decision maker with a moderate or high investment (Figure 5Bi). However, for a risk‐averse decision maker, the same prioritization and target probabilities (i.e., *Epicenter* with target probabilities of 0.5 and 0.75 for *p* and ϵ), but without community science data, was the best alternative for moderate or high investments (Figure 5Bii, Table 3). Under a low investment, *Linear* prioritization with target probabilities of 0.5 and 0.75 for *p* and ϵ was preferred for a risk‐neutral decision maker and *Epicenter* prioritization with target probabilities of 0.25 and 0.75 for *p* and ϵ was preferred for a risk‐averse decision maker.

Although the specific prioritization action may differ across invasion conditions, risk tolerances, and investment levels, the optimal target and detection probabilities were primarily 0.5 for *p* and 0.75 for ϵ (Figure 5, Table S2, Figures S2 and S3). Additional insight into the relationship between hours of effort and detection and eradication probabilities is presented in Figure S4. We found that High Invasion prioritization was never the sole preferred prioritization strategy, and No removals consistently performed poorly (Figure 5, Table S2).

**FIGURE 6:**
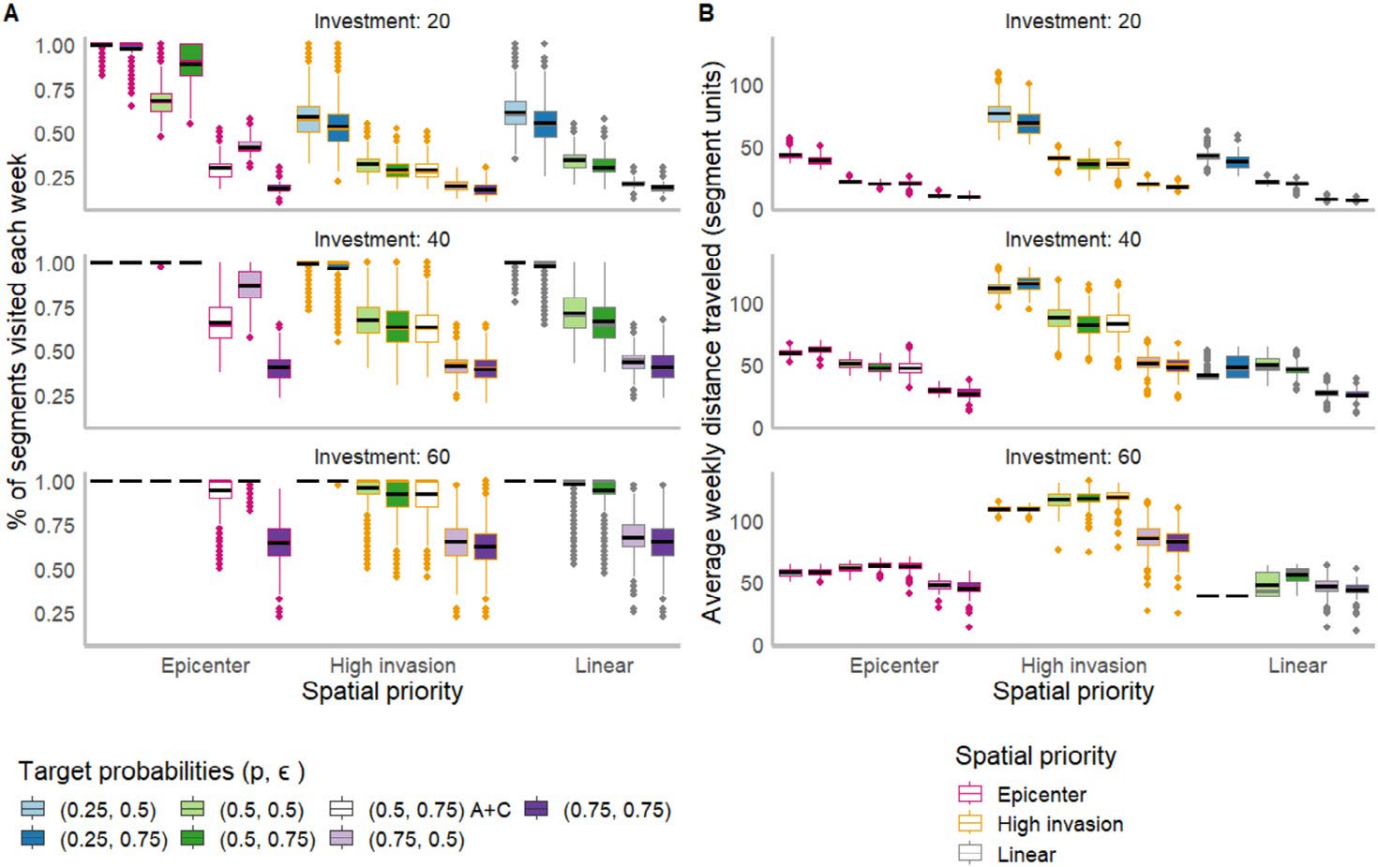
(A) Results of average percentage of segments visited for either detection or detection and removal for each alternative and investment level and (B) average weekly distance traveled (in terms of segment level units) for an emerging invasion (condition 2) for each alternative and investment level. In both plots, the boxplots represent outcomes from each alternative, the outline color of each box plot represents the spatial priority action, and the fill color represents the target (detection and eradication) probabilities. The alternatives with target priorities: (0.5, 0.75) A + C are the alternatives with the addition of community science data. In each boxplot, the colored line represents the median value, the black line is the mean value, the box displays the interquartile range, the lines indicate variability beyond the first and third quartiles, and the points represent outliers (See Figure S5 for outcomes under an established initial invasion).

Overall, we found that alternatives with *Epicenter* and *Linear* prioritizations allowed the manager to visit the most segments with a relatively low average cumulative distance traveled each week (Figure 6). In addition, *High Invasion* prioritization had the largest weekly distance traveled compared to the other prioritization approaches (Figure 6), since in this approach sites may be traveled through without being visited for management or monitoring. It was also evident that the weekly percentage of segments visited, and the weekly distance traveled, declined with increasing target detection (*p*) and eradication (ϵ) probabilities (Figure 6); this was predictable, as increasing effort at individual sites would necessarily reduce the time available for traveling between sites and the number of sites visited. These results were consistent across both sets of invasion conditions (Figure S5).

**FIGURE 7:**
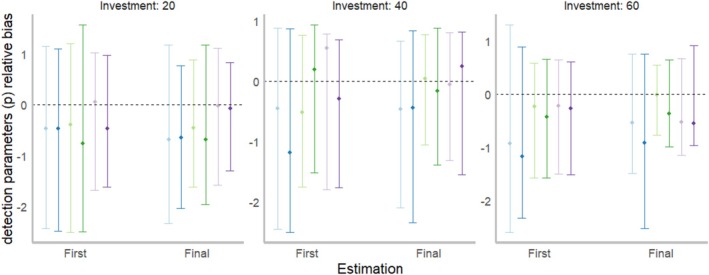
Results of relative bias estimates for detection parameters *p* for the high invasion prioritization under the established invasion (condition 1) for the three investment levels in terms of the first distinct year across alternatives (i.e., year 4) and final time the estimation model was run (i.e., year 10). The colors represent the different target probability pairings (*p* = detection, ϵ = eradication). The points represent average values across parameter sets and the error bars represent the upper and lower 5% quantile values.

Over time, we found that parameter and invasion state estimates improved under more than half of the management alternatives due to our Bayesian updating process (Figure 7, Figures S6–S11). However, we found that the relationship between management outcomes and statistical model performance was generally weak; the best‐performing alternative in each situation (i.e., for a given management investment, invasion condition, and decision criterion) was rarely the alternative that demonstrated the lowest relative bias (Table S2). For instance, although *Epicenter* with target probabilities of 0.5 and 0.75, for *p* and ϵ, respectively, was generally the best alternative in terms of expected value, this alternative did not have the lowest bias for invasion state, detection, or eradication parameter estimates (Table 3, Table S2). In addition, although the addition of community science data does not always benefit management outcomes, the addition did improve detection parameter estimates (Table S3). Across all alternatives, we found that on average, our model overestimated the invasion state and underestimated eradication parameters (Tables S2 and S3).

## 4 Discussion

Since invasive species management is often characterized by uncertainty, adaptive management is a powerful tool for identifying effective strategies for controlling these species. Using an adaptive management framework based on management strategy evaluation, we analyzed alternatives that were a function of location prioritization (i.e., the order in which segments were visited), effort per segment (through different target detection and eradication probabilities), investment level (total budgeted hours), and monitoring data types (including or excluding community science data), using invasive flowering rush in the Columbia River, USA, as a case study. We assessed alternatives under two invasion conditions: an established invasion (long invasion history) and an emerging invasion (short invasion history). From our management simulations, we identified four key lessons with implications for invasive species management.

The first key lesson is that alternatives involving learning, which allowed areas of high invasion to be prioritized, were commonly preferred for both emergent and established invasions (i.e., *Epicenter* and *High invasion* prioritizations, Figure 5, Table 3, Table S2). Hence, alternatives involving learning and adaptation outperformed alternatives in which the management strategy was static (i.e., *Linear* prioritization), a finding that is supported by research results in other conservation contexts (e.g., Canessa et al. 2016). In invasive species management, this result supports the benefit of both adaptive management and designing early detection and rapid response (EDRR) protocols, as they enable ongoing feedback to improve management and assess the invasion process (Bogich et al. 2008; Reaser et al. 2020). Previous research has shown that adaptive management and EDRR protocols can lead to favorable management outcomes. For example, in the context of an established invasion, Rout et al. (2017) showed that invasive plant management outcomes improved under an adaptive search method (i.e., visit locations with high invasion uncertainty), compared to a uniform search method to identify and remove invasive plants. In the context of emergent invasions, Sepulveda et al. (2023) found that eDNA tools for detecting invasive brittle naiad ( *Najas minor* ) were especially useful in lake environments with high invasion potential, and Marchessaux et al. (2023) suggested that prompt implementation of EDRR methods (e.g., integrating immediate overfishing measures) could reduce the risk of invasive blue crab populations (*Callinectes sapudus* and *Portunus segnis*) establishing in the Mediterranean Sea. Our findings bolster recent calls for increased use of adaptive management to improve outcomes in invasive species management (van Poorten and Beck 2021; Germino et al. 2022; Bunnell et al. 2023).

The second key lesson is that the integration of community science data was generally the most useful for risk‐neutral decision makers and was especially useful for emerging invasions (Figure 5, Table 3). We found that the integrated models with community science data often overestimated invasion (i.e., relative bias was higher than alternatives without community science data, Table 3 and Table S2). Overestimation may have occurred because community science data constituted binary observations (detected or not) while the underlying state process was a multistate model including three invasion severity states, and it is difficult to decipher the true invasion severity based on detection/nondetection data. The overestimation also led to fewer sites being visited for monitoring and management compared to models without community science data (Figure 6) because more time was spent at locations where invasion risk was believed to be high than at other segments. Hence, a risk‐averse decision maker may not benefit from community science data because it could lead to excessive time spent managing locations perceived as high‐risk for invasion when, in reality, invasion severity was low. Because the risk‐averse decision maker is looking for the alternative that presents the least risk for a particularly poor outcome (i.e., based on the mini–max criterion), it is better to be uncertain—and to select a strategy that is robust to that uncertainty—than to be wrong. For a risk‐neutral decision maker, the risk of being wrong is not as serious. This finding reveals the importance of assessing the accuracy of community science‐based observations when considering the role of community science data in invasive species management decisions (De Groot et al. 2022; Pocock et al. 2024; Gervazoni et al. 2023) and potentially integrating strategies to improve community scientist observations, such as volunteer trainings. This finding also emphasizes the importance of integrating risk attitude when managing under uncertainty (Runge and Converse 2020).

The third key lesson was that simulation outcomes favored maximum effort towards eradication but only moderate effort towards detection of flowering rush (Figure 5 and Table S2). The target detection and eradication probabilities that were most often optimal were, respectively, *p* = 0.5 and ϵ = 0.75. For eradication probability, this was the maximum value that we considered, but for detection probability, it was not. Under a lower target detection probability, less time was devoted to searching for flowering rush in any given segment (Figure S4) and more time was available to search and remove in more segments (Figure 6). Other resource allocation studies support the result that, under a constrained budget, more time spent on removal relative to monitoring will lead to improved management outcomes (Rout et al. 2014; Moore and McCarthy 2016; Liu et al. 2021). For example, Yemshanov et al. (2019) found that, for invasive emerald ash borer ( *Agrilus planipennis* ), the optimal management for population suppression involved visiting more sites for management and investing in cheaper, less effective, monitoring approaches. In another example, Rout et al. (2014) developed a modeling framework to determine allocation of management and surveillance for invasive black rats ( *Rattus rattus* ) in Australia and showed that it was better to manage under uncertainty regarding the presence of rats than to spend substantial resources confirming the presence of rats. Managers with an objective to suppress an invasive population might consider strategies involving more removal compared to strategies involving additional monitoring.

The final key lesson was that there is no clear positive relationship between optimal management outcomes and unbiased parameter estimates (Table 3, Tables S2 and S3). In fact, the alternative that performed the best in terms of suppressing the invasive population was often the alternative that performed the worst in terms of relative bias for invasion and parameter estimates (e.g., *Epicenter* prioritization with target probabilities of *p* = 0.5 and ϵ = 0.75; Table 3, Table S2). This outcome is related to the key lesson discussed previously, that more effort put into management is generally better than more effort put into monitoring: in occupancy modeling, estimates will generally improve as detection probabilities improve (Guillera‐Arroita et al. 2010). This outcome contradicts other studies that suggest that uncertainty in parameter values is a major impediment to invasive species management (Li et al. 2021; Pepin et al. 2022). We note, however, that our results are highly context dependent. For instance, for the case of invasive knotweed management ( *Fallopia japonica* ), managers acknowledged that instead of spending resources on studies to improve knowledge on knotweed invasion rates, these resources were better allocated to management (Cottet et al. 2015). However, Baxter and Possingham (2011) suggested that invasive red fire ant management ( *Solenopsis invicta* ) could benefit from learning about spread rates prior to management. Hence, to identify the importance of obtaining new information, applying value of information methods, which evaluate the difference between management outcomes with and without new information, is critical (Runge et al. 2011; Canessa et al. 2015; Healy et al. 2024).

Although we found that integrating community science data rarely improves outcomes (Figure 5, Table 3), there may be ancillary benefits in terms of informing the public about invasive species (Encarnação et al. 2021; Phillips et al. 2021; Compagnone et al. 2023). It was shown by Nanayakkara et al. (2018) that natural resource stakeholders in Saskatchewan, Canada, were not aware of the negative consequences of flowering rush. Therefore, a public education component to flowering rush management could help prevent flowering rush from establishing in new areas via human dispersal (Columbia Basin CWMA 2019). In addition, in a review, Anđelković et al. (2022) found that volunteer invasive species management programs can inspire greater commitment to protecting native species and their habitats. Hence, including a community science component in a removal program can, at a minimum aid in education about invasive species, which may reduce invasive species spread (Crall et al. 2013).

We demonstrated that adaptive management is an effective approach for invasive species management, despite its infrequent use in this field (Williams and Brown 2014; Lewandoski et al. 2021). The reasons for limited applications of adaptive management are many‐fold, including institutional skepticism, resources needed to collect and maintain monitoring data, and because the analytical approaches needed to identify optimal adaptive strategies are complex, not easily customized, time‐consuming, and require specialized quantitative skills (Westgate et al. 2013; Richardson et al. 2020; Nicol et al. 2022). However, adaptive management could be useful across various invasion establishment scenarios, wherein management and monitoring alternatives could be updated based on new information about the system (Figures 2 and 7). In addition, we attempted to overcome analytical adaptive management barriers by providing an approach based on management strategy evaluation that can be customized for a variety of invasion contexts.

Future studies could improve upon our modeling and adaptive management approach. First, the parameters describing biological and management processes for flowering rush in this region are unknown, and we had to rely on using “vague” priors for our initial estimation models (Table S1). Future studies could benefit from the addition of experiments and surveys to inform the initial parameter priors used in models (e.g., studies to understand biomass growth and dispersal). Second, we only accounted for a small range of target levels of detection and eradication probabilities, and we did not account for all possible control strategies for flowering rush. Combining mechanical hand removals with chemical treatments is an effective management approach (Turnage et al. 2019); however, chemical treatments are currently limited in this region due to management being conducted on National Wildlife Refuges (Columbia Basin CWMA 2019). In the future, biocontrol agents may be used in the region (Columbia Basin CWMA 2019), and future‐modeling methods could account for this additional management method. Third, we assumed that community scientists would not remove flowering rush, even if detected. In fact, without training, flowering rush may be removed incorrectly, producing plant fragments that can facilitate further spread (Johnson et al. 2009). If volunteers are trained for flowering rush removal, management outcomes may improve with this additional source of removal effort. In addition, although we assumed community scientists had lower detection probabilities for flowering rush, future studies could incorporate cases of false‐positive detections.

Identifying optimal allocation of effort towards monitoring and management is key to efficient use of conservation resources (Koch et al. 2020; Nguyen et al. 2024). We provide a framework to test management and monitoring approaches in an adaptive management context, wherein management can be iteratively updated through time as monitoring and learning occur.

Our results indicate that management approaches involving learning generally outperformed nonadaptive strategies, which provide strong support for future implementations of adaptive management.

## Author Contributions

**Brielle K. Thompson:** conceptualization (equal), data curation (lead), formal analysis (lead), funding acquisition (supporting), investigation (equal), methodology (equal), project administration (supporting), resources (supporting), software (lead), supervision (supporting), validation (equal), visualization (equal), writing – original draft (lead), writing – review and editing (equal). **Sarah J. Converse:** conceptualization (equal), data curation (supporting), formal analysis (supporting), funding acquisition (lead), investigation (equal), methodology (equal), project administration (lead), resources (lead), software (supporting), supervision (lead), validation (equal), visualization (equal), writing – original draft (supporting), writing – review and editing (equal). **Julian D. Olden:** conceptualization (equal), data curation (supporting), formal analysis (supporting), funding acquisition (lead), investigation (equal), methodology (equal), project administration (lead), resources (lead), software (supporting), supervision (lead), validation (equal), visualization (equal), writing – original draft (supporting), writing – review and editing (equal).

## Conflicts of Interest

The authors declare no conflicts of interest.

## Supporting information


Appendix S1.

## Data Availability

R scripts underpinning the analysis of this paper are publicly available on GitHub (https://github.com/Quantitative‐Conservation‐Lab/Thompson_etal_2025_EcologyandEvolution) and at Zenodo (https://doi.org/10.5281/zenodo.15078144). References to parameter values used in the model and supplemental figures and tables can be found in Appendix S1.
