# Correction to “Balancing Monitoring and Management in the Adaptive Management of an Invasive Species”

**DOI:** 10.1002/ece3.71446

**Published:** 2025-06-09

Thompson, B. K., Olden, J. D., & Converse, S. J. Balancing Monitoring and Management in the Adaptive Management of an Invasive Species. *Ecology and Evolution*. 2025; *15*(4) e71176.

In Figure 5 in the Results section, the legend for spatial priority was incorrect. We incorrectly described that: circle points indicate E = Epicenter, square points indicate H = High invasion, and triangle points indicate L = Linear.

The correct legend for spatial priority in Figure 5 is as follows: triangle points indicate E = Epicenter, circle points indicate H = High invasion, and square points indicate L = Linear. The correct version of Figure 5 is shown below.

**FIGURE 5:**
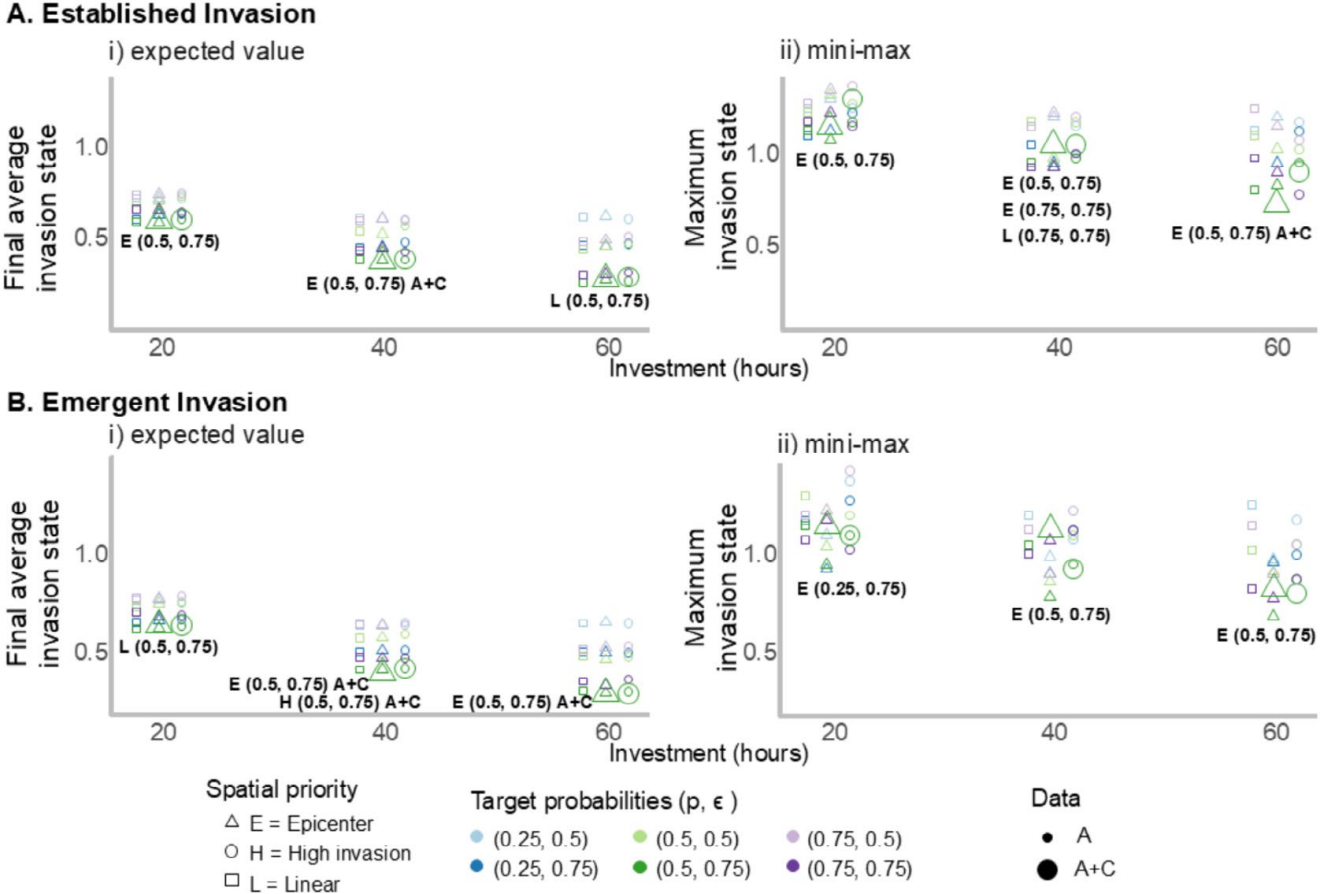
Performance of each simulated alternative in meeting the management objective to minimize the final average invasion state of flowering rush in the Columbia River study area, displayed across the three investment levels (20, 40, and 60h of effort per week). A final average invasion state of 0 indicates eradication while a final average invasion state of 2 indicates that all segments are in the high invasion state. In box A, outcomes are displayed for an established invasion, and in box B, outcomes are displayed for an emerging invasion. In plots A and B, subplot (i) displays the expected‐value outcome for each alternative across simulations for each investment level; the bolded text displays the alternative that performed best in terms of expected value. The expected‐value criterion is used for risk‐neutral decision makers. In plots A and B, subplot (ii) displays the maximum outcome for each alternative across simulations and for each investment level, the bolded text displays the alternative that performed best in terms of minimizing the maximum potential invasion outcome, or the “mini–max” criterion. This criterion is used for risk‐averse decision makers. The alternatives are a function of spatial priority (shown with the shapes), target detection and eradication probabilities (shown with colors), and the data used in estimation (shown with the size of the points) where “A” indicates just agency were collected, and “A+C” indicates agency and community science data were collected. In the bolded text, we indicate whether the top alternative included community science data with “A+C.”

We apologize for this error.

