# Supplementary material for: Balancing Monitoring and Management in the Adaptive Management of an Invasive Species
Source: Ecol Evol. 2025 Apr 1;15(4):e71176. doi: 10.1002/ece3.71176 (PMC11961554; doi:10.1002/ece3.71176)
Supplement: Supplementary file 1 — Appendix S1. [file ECE3-15-e71176-s001.docx]

**Appendix I: Additional Tables and Figures**


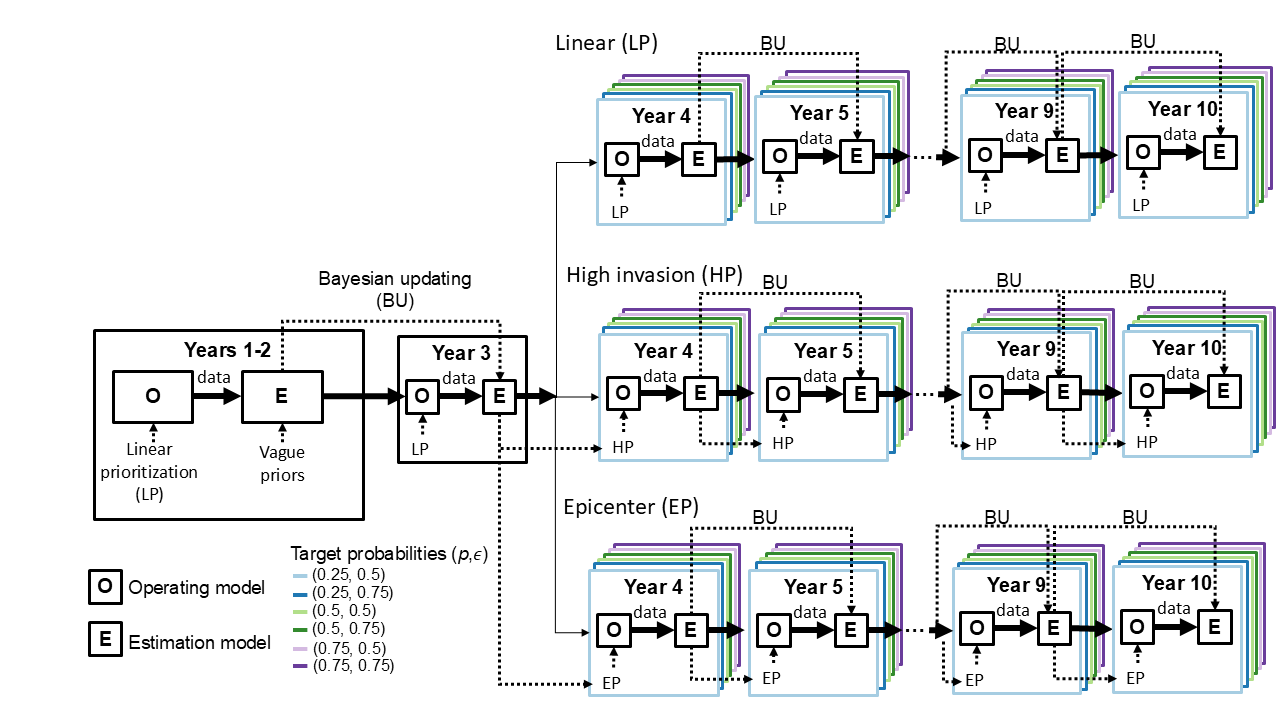


**Figure S1** Depiction of the management strategy evaluation (MSE) process for this study. The O boxes represent the operating model, and the E boxes represent the estimation model. During the first three years, removal locations were prioritized linearly, removal and search hours were randomized, the estimation model was fit using simulated monitoring data, and after year 2, Bayesian updating was used to update priors in the estimation model. After year 3, the Linear (LP), High invasion (HP), and Epicenter (EP) spatial priority approaches were tested under the six target probability pairs, indicated by the different colored boxes, where removal locations were prioritized according to the given approach and informed by results from the estimation model. Each operating and estimation model was run under 200 parameter sets to reflect different ecological and management assumptions.

**Table S1** Description of parameter distributions that were used to simulate the “true” parameter sets for the operating model. The values presented for each distribution value (e.g., mean, standard deviation, α and β shape parameters) were also the initial prior distributions, at the first time step, in the estimation model. See Table 2 in the main text for additional parameter details

| **Parameter** | **Description** | **Distribution** | **Rationale** (literature source) |
| --- | --- | --- | --- |
| $\beta_{0}^{[\epsilon,S]}$ | Logit-scale eradication probability for state *S* in the absence of management | *S = L*: Normal(-2, 0.5)  *S = H*: Normal(-3, 0.5) | Large infestations are likely to persist (Columbia Basin CWMA 2019) |
| $\beta_{1}^{[\epsilon,S]}$ | Logit-scale effect of removal effort on eradication probability | *S = L*: Normal(3, 0.5) > 0  *S = H*: Normal(2, 0.5) > 0 | Removal will lead to reduced biomass  (Columbia Basin CWMA 2019) |
| $\beta_{0}^{[\varphi,H]}$ | Logit-scale probability of staying in state *H* in the absence of management | Normal(2, 0.5) | Biomass growth is rapid (Gunderson et al. 2016) |
| $\beta_{1}^{[\varphi,H]}$ | Logit-scale effect of removal effort on the probability of staying in state H | Normal(1, 0.5), < 0 | Removal will lead to reduced biomass, but difficult to remove all fragments  (Columbia Basin CWMA 2019) |
| $\beta_{0}^{[\gamma]}$ | Logit-scale invasion probability in the absence of management | Normal(0, 0.5) | Highly suitable to many environments (Banerjee et al. 2020) |
| $\beta_{1}^{[\gamma]}$ | Logit-scale effect of habitat on invasion probability | Normal(1, 0.5) | Habitat dependent invasion (Madsen et al. 2016; Gebhart and Wersal 2024) |
| $\beta_{2}^{[\gamma]}$ | Logit-scale effect of adjacent invasion state on invasion probability | Normal(2, 0.5) | Neighboring propagule size leads to high invasion (Carter et al. 2018) |
| $d^{[S]}$ | Eradication probability for state *S* from the end of one data period to the start of the next data period (between data periods) | *S = L*: Beta(2, 15)  *S = H*: Beta(1, 20) | Biomass can remain during the non-growing season (Columbia Basin CWMA 2019). During growing season, growth is rapid (Gunderson et al. 2016) |
| $r^{[S]}$ | Probability of remaining in state *S* between data periods | *S = L*: Beta(2, 15)  *S = H*: Beta(14, 6) | Biomass can remain during the non growing season (Columbia Basin CWMA 2019). During growing season, growth is rapid (Gunderson et al. 2016) |
| $g$ | Probability of being in state *H* if invaded between data periods | Beta(12,12) | Rhizome density is highly variable (Madsen et al. 2012) |
| $\beta_{0}^{[p,S,A]}$ | Logit-scale detection probability for state *S* and Agency data, *A,* with no search effort | *S = L*: Normal(0, 0.5)  *S = H*: Normal(0, 0.5) | Detection is low (Columbia Basin CWMA 2019) |
| $\beta_{1}^{[p,S,A]}$ | Logit-scale effect of log(search effort) on detecting state *S* for agency data, *A* | *S = L*: Normal(2, 0.5) > 0  *S = H*: Normal(3, 0.5) > 0 | Higher invasions are easier to detect (Columbia Basin CWMA 2019) |
| $\delta$ | Probability of correctly observing state *H* | Beta(1, 1) | Certainty of invasion status is variable (Columbia Basin CWMA 2019) |
| $\beta_{0}^{[p,S,C]}$ | Logit-scale detection probability for state *S* and community science data, *C*, with no search effort | *S = L*: Normal(0, 0.5)  *S = H*: Normal(0, 0.5) | Detection is low (Columbia Basin CWMA 2019) |

**References for Table S1:**

Banerjee, A. K., Harms, N. E., Mukherjee, A., & Gaskin, J. F. (2020). Niche dynamics and potential distribution of Butomus umbellatus under current and future climate scenarios in North America. *Hydrobiologia*, *847*(6), 1505–1520. <https://doi.org/10.1007/s10750-020-04205-1>

Carter, C., Madsen, J. D., & Ervin, G. N. (2018). Effects of initial propagule size and water depth on Butomus umbellatus L. growth and vegetative propagation. *Aquatic Botany*, *150*, 27–32. <https://doi.org/10.1016/j.aquabot.2018.06.003>

Columbia Basin Cooperative Weed Management Area. (2019). *Columbia Basin Flowering Rush Management Plan: A regional strategy to address Butomus umbellatus throughout the Columbia Basin* (p. 67).

Gebhart, M. G., & Wersal, R. M. (2023). Ecological niche modeling of diploid flowering rush (*Butomus umbellatus* L.) in the United States. *Journal of Freshwater Ecology*, *39*(1), 2292232. <https://doi.org/10.1080/02705060.2023.2292232>

Gunderson, M., Kapuscinski, K., Crane, D., & Farrell, J. (2016). Habitats colonized by non-native flowering rush Butomus umbellatus (Linnaeus, 1753) in the Niagara River, USA. *Aquatic Invasions*, *11*(4), 369–380. <https://doi.org/10.3391/ai.2016.11.4.03>

Madsen, J. D., Wersal, R. M., Marko, M. D., & Skogerboe, J. G. (2012). Ecology and management of flowering rush (Butomus umbellatus) in the Detroit Lakes, Minnesota (5054; Geosystems Research Institute Report).

Madsen, J. D., Sartain, B., Turnage, G., & Marko, M. (2016). Management of flowering rush in the Detroit Lakes, Minnesota. *J. Aquat. Plant Manage.*

**Table S2** Results for every alternative evaluated for invasive flowering rush control, which are functions of investment, spatial priority, and target detection and eradication probabilities (denoted as Target Probabilities). We display results first for an established invasion (condition 1), followed by results for an emergent initial invasion (condition 2). For each investment level, the best-performing alternative and associated outcome are colored in green, and the worst is colored in gray. The results include the mean and maximum outcome in the suppression objective (final average invasion state), and mean outcome in relative bias and Root Mean Square Error for invasion state estimate (State rel bias and State RMSE), detection (*p* rel. bias and *p* RMSE) and eradication (ϵ rel bias and ϵ RMSE) probabilities.

|  |  |  | Objective | | Model performance | | | | | |
| --- | --- | --- | --- | --- | --- | --- | --- | --- | --- | --- |
| Alternative | | | Suppression  *aim to minimize* | | State rel. bias | State RMSE | *p* rel. bias | *p*  RMSE | ϵ rel. bias | ϵ RMSE |
| **Investment** | **Spatial priority** | **Target Probabilities (*p*, ϵ)** | **Mean** | **Max** | **Mean** | **Mean** | **Mean** | **Mean** | **Mean** | **Mean** |
| ***Established Invasion (Condition* 1)** | | | | | | | | | | |
| None (0) | None | (0,0) | 0.934 | 1.3 | NA | NA | NA | NA | NA | NA |
| Low  (20) | *High invasion* | (0.25, 0.5) | 0.765 | 1.250 | 0.214 | 0.677 | -0.628 | 0.372 | -0.074 | 0.502 |
|  |  | (0.25, 0.75) | 0.668 | 1.230 | 0.232 | 0.673 | -0.579 | 0.374 | -0.081 | 0.502 |
|  |  | (0.5, 0.5) | 0.754 | 1.270 | 0.163 | 0.595 | -0.365 | 0.364 | -0.070 | 0.485 |
|  |  | (0.5, 0.75) | 0.638 | 1.180 | 0.166 | 0.608 | -0.589 | 0.385 | -0.071 | 0.486 |
|  |  | (0.75, 0.5) | 0.782 | 1.380 | 0.134 | 0.554 | 0.050 | 0.360 | -0.067 | 0.497 |
|  |  | (0.75, 0.75) | 0.675 | 1.150 | 0.140 | 0.575 | -0.138 | 0.353 | -0.068 | 0.456 |
|  | *Linear* | (0.25, 0.5) | 0.753 | 1.250 | 0.224 | 0.669 | -0.392 | 0.359 | -0.076 | 0.503 |
|  |  | (0.25, 0.75) | 0.636 | 1.100 | 0.229 | 0.667 | -0.656 | 0.372 | -0.085 | 0.509 |
|  |  | (0.5, 0.5) | 0.727 | 1.230 | 0.164 | 0.579 | -0.101 | 0.357 | -0.064 | 0.488 |
|  |  | (0.5, 0.75) | 0.618 | 1.120 | 0.167 | 0.601 | -0.898 | 0.375 | -0.066 | 0.498 |
|  |  | (0.75, 0.5) | 0.772 | 1.270 | 0.130 | 0.531 | 0.144 | 0.353 | -0.063 | 0.495 |
|  |  | (0.75, 0.75) | 0.691 | 1.180 | 0.135 | 0.545 | 0.017 | 0.355 | -0.063 | 0.451 |
|  | *Epicenter* | (0.25, 0.5) | 0.756 | 1.300 | 0.221 | 0.673 | -0.703 | 0.361 | -0.078 | 0.508 |
|  |  | (0.25, 0.75) | 0.660 | 1.120 | 0.225 | 0.668 | -0.391 | 0.375 | -0.087 | 0.503 |
|  |  | (0.5, 0.5) | 0.737 | 1.320 | 0.160 | 0.585 | -0.184 | 0.358 | -0.067 | 0.485 |
|  |  | (0.5, 0.75) | 0.617 | 1.080 | 0.175 | 0.603 | -0.265 | 0.378 | -0.069 | 0.492 |
|  |  | (0.75, 0.5) | 0.778 | 1.350 | 0.127 | 0.542 | 0.048 | 0.353 | -0.065 | 0.495 |
|  |  | (0.75, 0.75) | 0.691 | 1.230 | 0.140 | 0.556 | 0.216 | 0.365 | -0.064 | 0.455 |
| Moderate  (40) | *High invasion* | (0.25, 0.5) | 0.635 | 1.150 | 0.201 | 0.580 | -0.439 | 0.344 | -0.129 | 0.569 |
|  |  | (0.25, 0.75) | 0.510 | 1.000 | 0.222 | 0.554 | -0.605 | 0.363 | -0.166 | 0.622 |
|  |  | (0.5, 0.5) | 0.600 | 1.180 | 0.122 | 0.407 | -0.080 | 0.317 | -0.142 | 0.561 |
|  |  | (0.5, 0.75) | 0.416 | 0.975 | 0.129 | 0.365 | 0.046 | 0.334 | -0.172 | 0.608 |
|  |  | (0.75, 0.5) | 0.632 | 1.200 | 0.071 | 0.368 | 0.029 | 0.333 | -0.109 | 0.517 |
|  |  | (0.75, 0.75) | 0.458 | 1.000 | 0.057 | 0.330 | 0.039 | 0.333 | -0.113 | 0.455 |
|  | *Linear* | (0.25, 0.5) | 0.641 | 1.150 | 0.198 | 0.576 | -0.306 | 0.354 | -0.125 | 0.571 |
|  |  | (0.25, 0.75) | 0.483 | 1.050 | 0.213 | 0.546 | -0.604 | 0.373 | -0.167 | 0.622 |
|  |  | (0.5, 0.5) | 0.572 | 1.180 | 0.139 | 0.398 | -0.314 | 0.322 | -0.145 | 0.568 |
|  |  | (0.5, 0.75) | 0.416 | 0.950 | 0.131 | 0.364 | -0.438 | 0.333 | -0.178 | 0.625 |
|  |  | (0.75, 0.5) | 0.630 | 1.150 | 0.065 | 0.355 | -0.059 | 0.335 | -0.109 | 0.516 |
|  |  | (0.75, 0.75) | 0.466 | 0.925 | 0.058 | 0.327 | 0.083 | 0.335 | -0.110 | 0.455 |
|  | *Epicenter* | (0.25, 0.5) | 0.640 | 1.200 | 0.207 | 0.576 | -0.447 | 0.350 | -0.127 | 0.574 |
|  |  | (0.25, 0.75) | 0.482 | 0.950 | 0.219 | 0.546 | -0.545 | 0.368 | -0.166 | 0.625 |
|  |  | (0.5, 0.5) | 0.558 | 0.975 | 0.137 | 0.400 | -0.094 | 0.325 | -0.145 | 0.562 |
|  |  | (0.5, 0.75) | 0.416 | 0.925 | 0.130 | 0.363 | -0.187 | 0.328 | -0.177 | 0.624 |
|  |  | (0.75, 0.5) | 0.639 | 1.230 | 0.073 | 0.360 | 0.122 | 0.337 | -0.113 | 0.520 |
|  |  | (0.75, 0.75) | 0.478 | 0.925 | 0.058 | 0.329 | -0.027 | 0.337 | -0.116 | 0.460 |
| High  (60) | *High invasion* | (0.25, 0.5) | 0.638 | 1.180 | 0.235 | 0.582 | -0.660 | 0.346 | -0.158 | 0.615 |
|  |  | (0.25, 0.75) | 0.504 | 1.120 | 0.234 | 0.550 | -0.997 | 0.360 | -0.194 | 0.659 |
|  |  | (0.5, 0.5) | 0.496 | 1.020 | 0.130 | 0.335 | -0.022 | 0.295 | -0.174 | 0.608 |
|  |  | (0.5, 0.75) | 0.299 | 0.950 | 0.121 | 0.263 | -0.401 | 0.302 | -0.208 | 0.676 |
|  |  | (0.75, 0.5) | 0.543 | 1.080 | 0.097 | 0.297 | -0.439 | 0.303 | -0.190 | 0.613 |
|  |  | (0.75, 0.75) | 0.344 | 0.775 | 0.089 | 0.246 | -0.521 | 0.315 | -0.213 | 0.572 |
|  | *Linear* | (0.25, 0.5) | 0.651 | 1.120 | 0.213 | 0.575 | -0.880 | 0.340 | -0.161 | 0.616 |
|  |  | (0.25, 0.75) | 0.496 | 0.975 | 0.235 | 0.548 | -0.680 | 0.356 | -0.192 | 0.659 |
|  |  | (0.5, 0.5) | 0.475 | 1.100 | 0.131 | 0.326 | -0.415 | 0.298 | -0.170 | 0.603 |
|  |  | (0.5, 0.75) | 0.290 | 0.800 | 0.115 | 0.261 | 0.057 | 0.299 | -0.210 | 0.675 |
|  |  | (0.75, 0.5) | 0.516 | 1.250 | 0.101 | 0.292 | -0.467 | 0.303 | -0.192 | 0.617 |
|  |  | (0.75, 0.75) | 0.329 | 0.975 | 0.106 | 0.255 | -0.767 | 0.323 | -0.214 | 0.593 |
|  | *Epicenter* | (0.25, 0.5) | 0.655 | 1.200 | 0.212 | 0.571 | -0.735 | 0.342 | -0.159 | 0.617 |
|  |  | (0.25, 0.75) | 0.489 | 0.950 | 0.231 | 0.544 | -0.546 | 0.358 | -0.192 | 0.658 |
|  |  | (0.5, 0.5) | 0.491 | 1.020 | 0.134 | 0.328 | -0.589 | 0.289 | -0.172 | 0.596 |
|  |  | (0.5, 0.75) | 0.309 | 0.825 | 0.117 | 0.261 | -0.418 | 0.297 | -0.214 | 0.683 |
|  |  | (0.75, 0.5) | 0.523 | 1.150 | 0.111 | 0.292 | -0.179 | 0.310 | -0.192 | 0.613 |
|  |  | (0.75, 0.75) | 0.335 | 0.900 | 0.095 | 0.246 | -0.415 | 0.318 | -0.215 | 0.589 |
| ***Emergent Invasion (Condition 2)*** | | | | | | | | | | |
| None (0) | *None* | (0,0) | 0.941 | 1.375 | NA | NA | NA | NA | NA | NA |
| Low  (20) | *High invasion* | (0.25, 0.5) | 0.753 | 1.380 | 0.213 | 0.675 | -0.648 | 0.372 | -0.057 | 0.465 |
|  |  | (0.25, 0.75) | 0.663 | 1.270 | 0.228 | 0.670 | -0.876 | 0.396 | -0.066 | 0.474 |
|  |  | (0.5, 0.5) | 0.745 | 1.200 | 0.159 | 0.589 | -0.720 | 0.373 | -0.051 | 0.460 |
|  |  | (0.5, 0.75) | 0.631 | 1.100 | 0.178 | 0.603 | -0.395 | 0.384 | -0.052 | 0.469 |
|  |  | (0.75, 0.5) | 0.784 | 1.420 | 0.130 | 0.549 | -0.358 | 0.362 | -0.047 | 0.465 |
|  |  | (0.75, 0.75) | 0.685 | 1.020 | 0.144 | 0.570 | -0.526 | 0.361 | -0.047 | 0.446 |
|  | *Linear* | (0.25, 0.5) | 0.760 | 1.180 | 0.231 | 0.664 | -0.711 | 0.371 | -0.061 | 0.473 |
|  |  | (0.25, 0.75) | 0.648 | 1.180 | 0.224 | 0.663 | -0.822 | 0.388 | -0.067 | 0.481 |
|  |  | (0.5, 0.5) | 0.724 | 1.300 | 0.160 | 0.569 | -0.801 | 0.366 | -0.047 | 0.458 |
|  |  | (0.5, 0.75) | 0.612 | 1.150 | 0.175 | 0.592 | -0.698 | 0.384 | -0.048 | 0.465 |
|  |  | (0.75, 0.5) | 0.774 | 1.200 | 0.139 | 0.526 | -0.317 | 0.359 | -0.043 | 0.464 |
|  |  | (0.75, 0.75) | 0.698 | 1.080 | 0.138 | 0.545 | -0.723 | 0.371 | -0.042 | 0.440 |
|  | *Epicenter* | (0.25, 0.5) | 0.762 | 1.100 | 0.218 | 0.669 | -0.428 | 0.378 | -0.060 | 0.471 |
|  |  | (0.25, 0.75) | 0.656 | 0.925 | 0.246 | 0.668 | -0.623 | 0.382 | -0.066 | 0.477 |
|  |  | (0.5, 0.5) | 0.742 | 1.040 | 0.161 | 0.576 | -0.724 | 0.371 | -0.048 | 0.459 |
|  |  | (0.5, 0.75) | 0.619 | 0.950 | 0.166 | 0.594 | -0.400 | 0.392 | -0.051 | 0.462 |
|  |  | (0.75, 0.5) | 0.768 | 1.230 | 0.128 | 0.530 | -1.280 | 0.357 | -0.048 | 0.461 |
|  |  | (0.75, 0.75) | 0.675 | 1.180 | 0.133 | 0.557 | -0.413 | 0.359 | -0.047 | 0.439 |
| Moderate  (40) | *High invasion* | (0.25, 0.5) | 0.648 | 1.080 | 0.197 | 0.577 | -0.054 | 0.346 | -0.117 | 0.546 |
|  |  | (0.25, 0.75) | 0.504 | 1.080 | 0.228 | 0.557 | -0.361 | 0.356 | -0.153 | 0.601 |
|  |  | (0.5, 0.5) | 0.587 | 1.100 | 0.134 | 0.409 | 0.159 | 0.311 | -0.129 | 0.550 |
|  |  | (0.5, 0.75) | 0.415 | 0.950 | 0.132 | 0.367 | -0.306 | 0.316 | -0.156 | 0.596 |
|  |  | (0.75, 0.5) | 0.634 | 1.230 | 0.072 | 0.367 | -0.496 | 0.326 | -0.097 | 0.500 |
|  |  | (0.75, 0.75) | 0.467 | 1.120 | 0.062 | 0.334 | 0.024 | 0.328 | -0.101 | 0.460 |
|  | *Linear* | (0.25, 0.5) | 0.642 | 1.200 | 0.192 | 0.577 | -0.026 | 0.351 | -0.119 | 0.545 |
|  |  | (0.25, 0.75) | 0.494 | 1.000 | 0.226 | 0.552 | -0.403 | 0.355 | -0.157 | 0.602 |
|  |  | (0.5, 0.5) | 0.566 | 1.120 | 0.129 | 0.392 | -0.134 | 0.311 | -0.133 | 0.548 |
|  |  | (0.5, 0.75) | 0.407 | 1.050 | 0.134 | 0.357 | -0.275 | 0.316 | -0.158 | 0.600 |
|  |  | (0.75, 0.5) | 0.635 | 1.120 | 0.067 | 0.351 | -0.173 | 0.324 | -0.095 | 0.503 |
|  |  | (0.75, 0.75) | 0.470 | 1.000 | 0.061 | 0.326 | -0.085 | 0.325 | -0.092 | 0.456 |
|  | *Epicenter* | (0.25, 0.5) | 0.635 | 0.988 | 0.198 | 0.579 | -0.267 | 0.347 | -0.118 | 0.545 |
|  |  | (0.25, 0.75) | 0.504 | 0.900 | 0.231 | 0.557 | -0.344 | 0.358 | -0.153 | 0.606 |
|  |  | (0.5, 0.5) | 0.570 | 0.862 | 0.132 | 0.398 | 0.292 | 0.307 | -0.136 | 0.550 |
|  |  | (0.5, 0.75) | 0.407 | 0.783 | 0.135 | 0.364 | -0.055 | 0.321 | -0.159 | 0.600 |
|  |  | (0.75, 0.5) | 0.630 | 0.900 | 0.070 | 0.355 | -0.120 | 0.319 | -0.098 | 0.495 |
|  |  | (0.75, 0.75) | 0.464 | 1.080 | 0.065 | 0.328 | 0.206 | 0.320 | -0.096 | 0.455 |
| High  (60) | *High invasion* | (0.25, 0.5) | 0.643 | 1.180 | 0.198 | 0.565 | -0.109 | 0.351 | -0.126 | 0.572 |
|  |  | (0.25, 0.75) | 0.492 | 1.000 | 0.209 | 0.529 | -0.101 | 0.358 | -0.161 | 0.622 |
|  |  | (0.5, 0.5) | 0.473 | 1.050 | 0.118 | 0.320 | 0.397 | 0.299 | -0.135 | 0.562 |
|  |  | (0.5, 0.75) | 0.297 | 0.875 | 0.103 | 0.325 | 0.050 | 0.311 | -0.175 | 0.636 |
|  |  | (0.75, 0.5) | 0.526 | 1.050 | 0.091 | 0.287 | 0.466 | 0.314 | -0.156 | 0.564 |
|  |  | (0.75, 0.75) | 0.357 | 0.875 | 0.092 | 0.242 | -0.325 | 0.317 | -0.177 | 0.526 |
|  | *Linear* | (0.25, 0.5) | 0.644 | 1.250 | 0.190 | 0.564 | -0.100 | 0.353 | -0.127 | 0.576 |
|  |  | (0.25, 0.75) | 0.494 | 1.020 | 0.205 | 0.530 | -0.275 | 0.356 | -0.162 | 0.628 |
|  |  | (0.5, 0.5) | 0.477 | 1.020 | 0.115 | 0.312 | 0.236 | 0.299 | -0.137 | 0.568 |
|  |  | (0.5, 0.75) | 0.296 | 0.825 | 0.106 | 0.244 | 0.094 | 0.309 | -0.174 | 0.633 |
|  |  | (0.75, 0.5) | 0.506 | 1.150 | 0.103 | 0.286 | -0.101 | 0.317 | -0.153 | 0.566 |
|  |  | (0.75, 0.75) | 0.348 | 0.825 | 0.097 | 0.244 | -0.157 | 0.317 | -0.183 | 0.544 |
|  | *Epicenter* | (0.25, 0.5) | 0.649 | 0.975 | 0.187 | 0.562 | -0.258 | 0.352 | -0.126 | 0.572 |
|  |  | (0.25, 0.75) | 0.496 | 0.962 | 0.208 | 0.533 | -0.266 | 0.369 | -0.164 | 0.627 |
|  |  | (0.5, 0.5) | 0.458 | 0.912 | 0.119 | 0.318 | 0.136 | 0.297 | -0.134 | 0.562 |
|  |  | (0.5, 0.75) | 0.287 | 0.683 | 0.108 | 0.255 | -0.061 | 0.307 | -0.180 | 0.643 |
|  |  | (0.75, 0.5) | 0.521 | 0.900 | 0.098 | 0.288 | -0.222 | 0.312 | -0.158 | 0.571 |
|  |  | (0.75, 0.75) | 0.330 | 0.775 | 0.096 | 0.242 | 0.208 | 0.316 | -0.179 | 0.532 |

|  |  |  |  | Model Performance | | | | |
| --- | --- | --- | --- | --- | --- | --- | --- | --- |
| Alternative | | | | State RMSE | p rel. bias | p  RMSE | ϵ rel. bias | ϵ  RMSE |
| **Investment**  (hours/week) | **Spatial priority** | **Target probabilities (*p*, ϵ)** | **Data** | **Mean**  **outcome** | **Mean**  **outcome** | **Mean**  **outcome** | **Mean**  **outcome** | **Mean**  **outcome** |
| ***Established Invasion (Condition* 1*)*** | | | | | | | | |
| Low  (20) | *High invasion* | (0.5, 0.75) | A | 0.608 | -0.589 | 0.385 | -0.071 | 0.486 |
|  |  | (0.5, 0.75) | A+C | 0.566 | 0.014 | 0.359 | -0.120 | 0.528 |
|  | *Epicenter* | (0.5, 0.75) | A | 0.603 | -0.265 | 0.378 | -0.069 | 0.492 |
|  |  | (0.5, 0.75) | A+C | 0.556 | -0.155 | 0.345 | -0.132 | 0.550 |
|  | *Linear* | (0.5, 0.75) | A | 0.601 | -0.898 | 0.375 | -0.066 | 0.498 |
| Moderate  (40) | *High invasion* | (0.5, 0.75) | A | 0.365 | 0.046 | 0.334 | -0.172 | 0.608 |
|  |  | (0.5, 0.75) | A+C | 0.354 | -0.014 | 0.338 | -0.210 | 0.663 |
|  | *Epicenter* | (0.5, 0.75) | A | 0.363 | -0.187 | 0.328 | -0.177 | 0.624 |
|  |  | (0.5, 0.75) | A+C | 0.346 | -0.008 | 0.325 | -0.222 | 0.678 |
|  | *Linear* | (0.5, 0.75) | A | 0.364 | -0.438 | 0.333 | -0.178 | 0.625 |
| High  (60) | *High invasion* | (0.5, 0.75) | A | 0.263 | -0.401 | 0.302 | -0.208 | 0.676 |
|  |  | (0.5, 0.75) | A+C | 0.270 | 0.0313 | 0.327 | -0.227 | 0.676 |
|  | *Epicenter* | (0.5, 0.75) | A | 0.261 | -0.418 | 0.297 | -0.214 | 0.683 |
|  |  | (0.5, 0.75) | A+C | 0.252 | -0.339 | 0.298 | -0.234 | 0.690 |
|  | *Linear* | (0.5, 0.75) | A | 0.261 | 0.057 | 0.299 | -0.210 | 0.675 |
| ***Emergent Invasion (Condition* 2*)*** | | | | | | | | |
| Low  (20) | *High invasion* | (0.5, 0.75) | A | 0.603 | -0.395 | 0.384 | -0.052 | 0.469 |
|  |  | (0.5, 0.75) | A+C | 0.557 | -0.069 | 0.357 | -0.115 | 0.531 |
|  | *Epicenter* | (0.5, 0.75) | A | 0.594 | -0.400 | 0.392 | -0.051 | 0.462 |
|  |  | (0.5, 0.75) | A+C | 0.553 | 0.261 | 0.354 | -0.116 | 0.533 |
|  | *Linear* | (0.5, 0.75) | A | 0.592 | -0.698 | 0.384 | -0.048 | 0.465 |
| Moderate  (40) | *High invasion* | (0.5, 0.75) | A | 0.367 | -0.306 | 0.316 | -0.156 | 0.596 |
|  |  | (0.5, 0.75) | A+C | 0.357 | 0.142 | 0.314 | -0.206 | 0.657 |
|  | *Epicenter* | (0.5, 0.75) | A | 0.364 | -0.055 | 0.321 | -0.159 | 0.600 |
|  |  | (0.5, 0.75) | A+C | 0.346 | 0.149 | 0.308 | -0.209 | 0.662 |
|  | *Linear* | (0.5, 0.75) | A | 0.357 | -0.275 | 0.316 | -0.158 | 0.600 |
| High  (60) | *High invasion* | (0.5, 0.75) | A | 0.325 | 0.050 | 0.311 | -0.175 | 0.636 |
|  |  | (0.5, 0.75) | A+C | 0.250 | 0.266 | 0.300 | -0.201 | 0.644 |
|  | *Epicenter* | (0.5, 0.75) | A | 0.255 | -0.061 | 0.307 | -0.180 | 0.643 |
|  |  | (0.5, 0.75) | A+C | 0.248 | -0.234 | 0.299 | -0.200 | 0.642 |
|  | *Linear* | (0.5, 0.75) | A | 0.244 | 0.094 | 0.309 | -0.174 | 0.633 |

**Table S3** Results of model performance under both invasion conditions for a subset of alternatives with the addition of community science data. We display results first for an established invasion (condition 1), followed by results for an emergent initial invasion (condition 2). We show the performance of the addition of community science data (noted by Data A + C) and without community science data (A) and compare outcomes against the best expected value result, Linear (0.5, 0.75). For each investment level, the best-performing alternative and associated outcome are colored in green, and the worst is colored in gray. The model performance outcomes identified from the model simulations included the Root Mean Square error for invasion state estimate (State RMSE), and mean outcome in relative bias and Root Mean Square error for detection (p rel. bias and p RMSE) and eradication (ϵ rel bias and ϵ RMSE) probabilities across simulations for each alternative. Each alternative was denoted by the spatial priority action, target detection and eradication probabilities (Target probabilities) whether community science data were included (Data), and the investment hours (Investment).


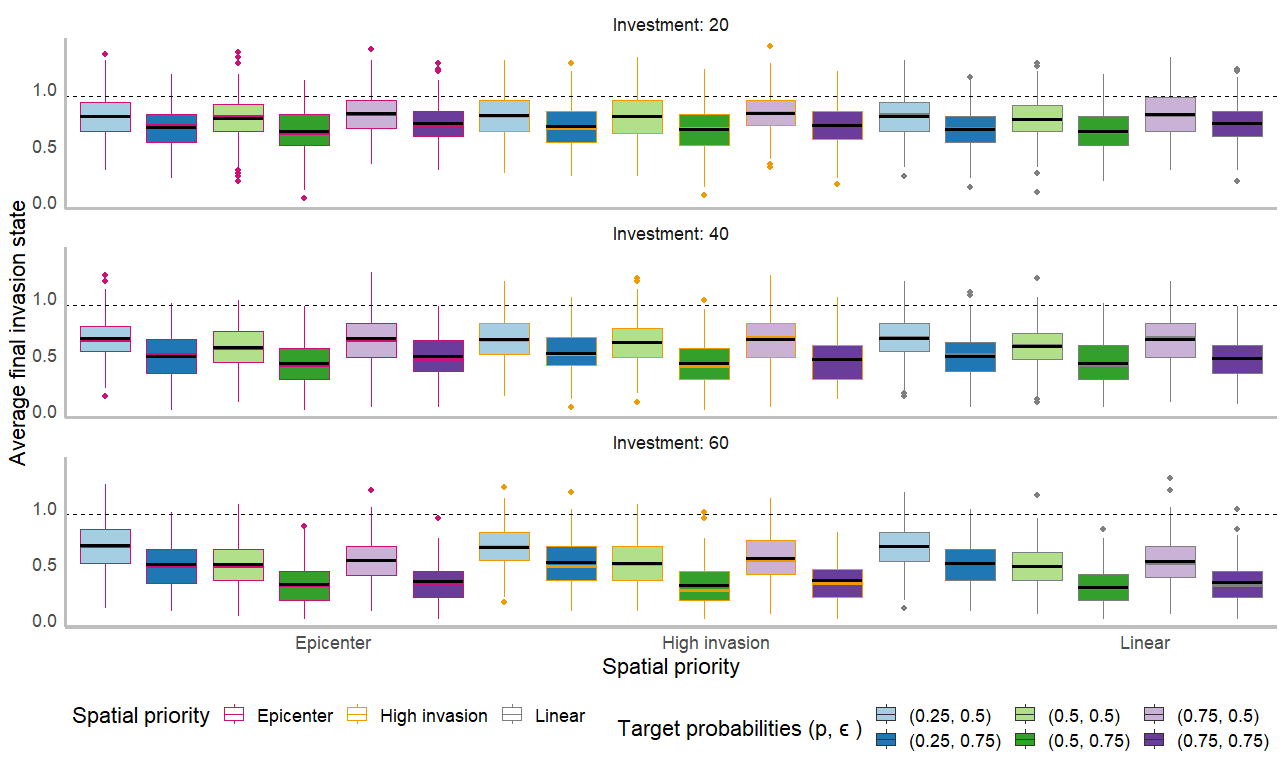


**Figure S2** Results of performance under the suppression objective for flowering rush control, for all alternatives under the three investment levels for an established initial invasion (condition 1). The boxplots represent outcomes from each alternative, the outline color of each box plot represents the spatial priority, and the fill color represents the target probability pairings. In each boxplot, the colored line represents the median value, the black line is the mean value, the boxplot displays the interquartile range, the lines indicate variability beyond the first and third quartiles, and the points represent outliers.


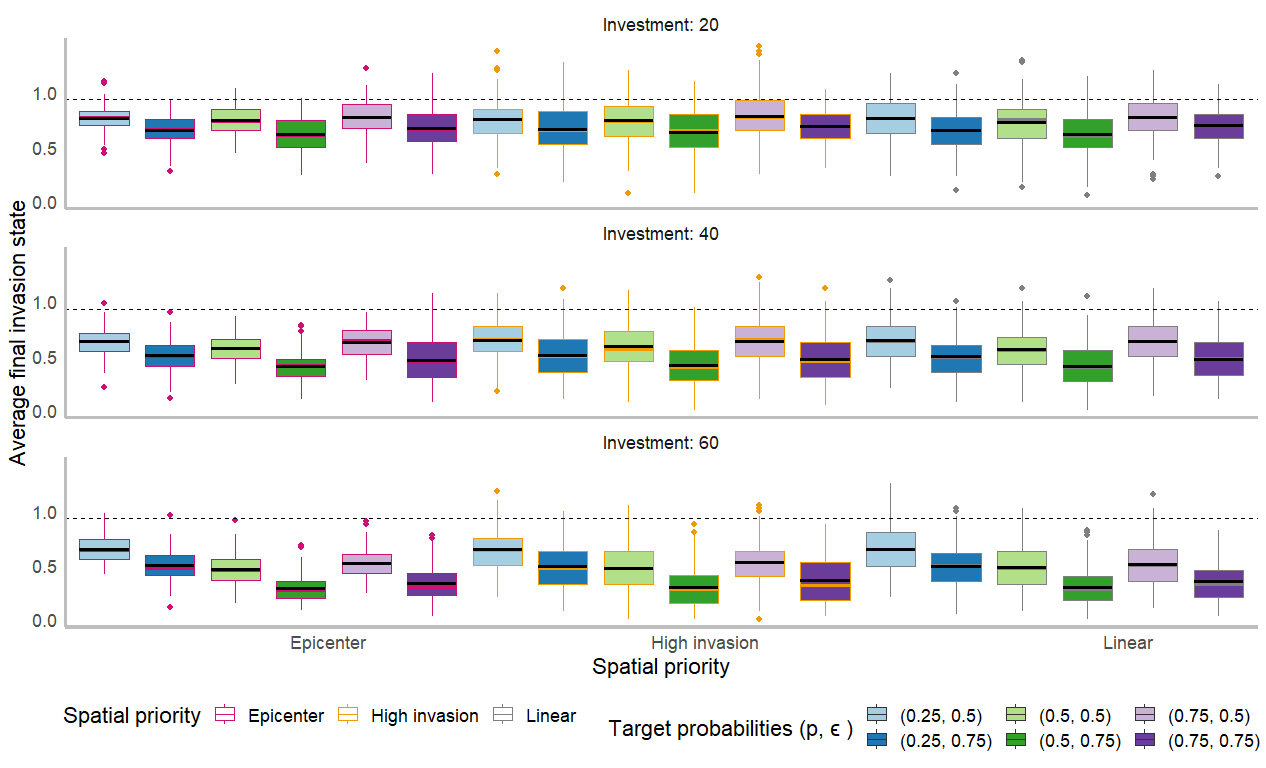


**Figure S3** Results of performance under the suppression objective for flowering rush control, for all alternatives under the three investment levels for an emerging initial invasion (condition 2). The boxplots represent outcomes from each alternative, the outline color of each box plot represents the spatial priority, and the fill color represents the target probability pairings. In each boxplot, the colored line represents the median value, the black line is the mean value, the boxplot displays the interquartile range, the lines indicate variability beyond the first and third quartiles, and the points represent outliers.


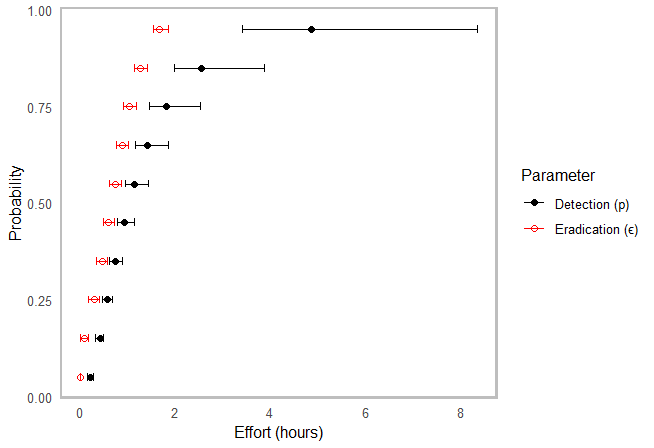


**Figure S4** The relationship between effort and target probabilities for detection and eradication. We display detection probabilities, *p*, with black closed circles, and eradication probability, $\epsilon$, with red open circles. The points represent average values across parameter sets, invasion states, investment levels, and the two initial invasion scenarios. The error bars represent the upper and lower 5% quantile values.


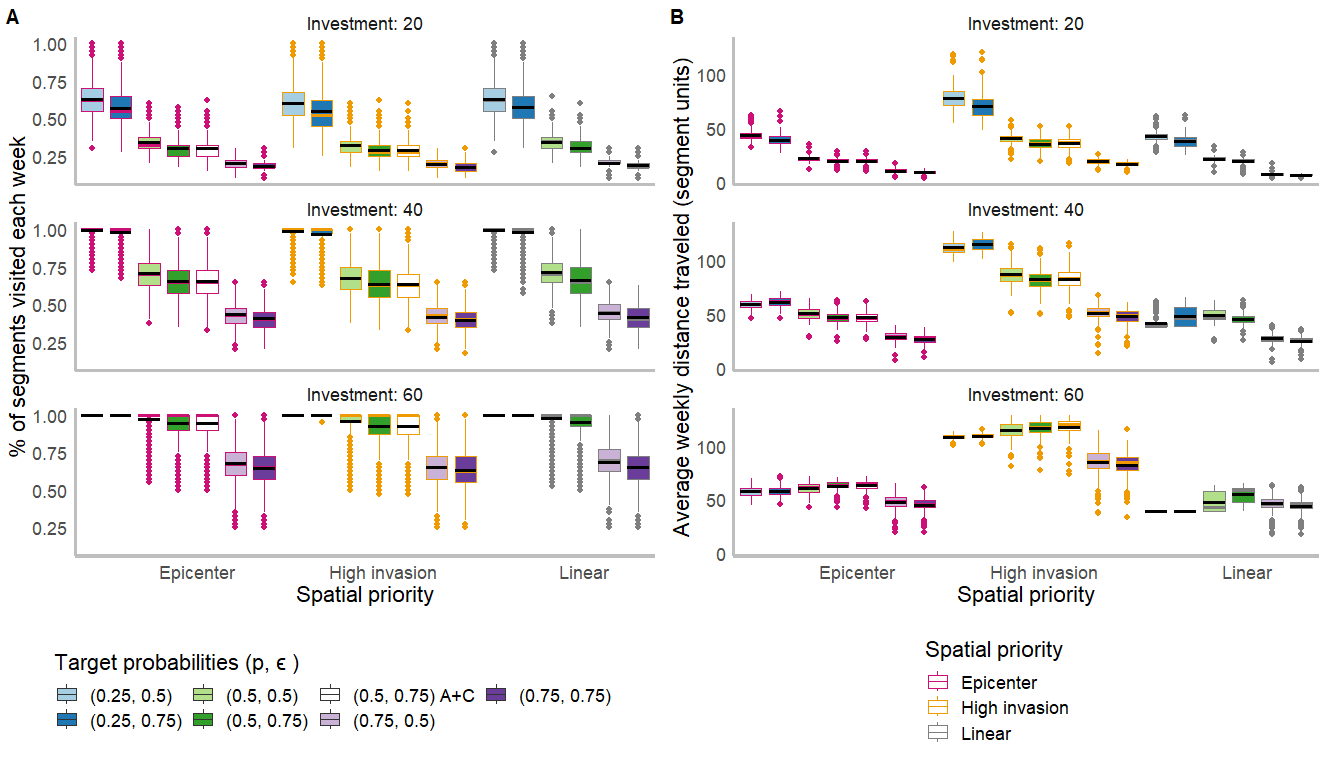


**Figure S5** A) Results of average percentage of segments that are visited for either detection or removal for each alternative and investment level and B) Average weekly distance traveled (in terms of segment level units) for an established invasion (condition 1) for each alternative and investment level. In both plots, the boxplots represent outcomes from each alternative, the outline color of each box plot represents the spatial priority, and the fill color represents the target probability pairings. The alternatives with target priorities: (0.5, 0.75) A + C are the alternatives with the addition of community science data. In each boxplot, the colored line represents the median value, the black line is the mean value, the boxplot displays the interquartile range, the lines indicate variability beyond the first and third quartiles, and the points represent outliers.


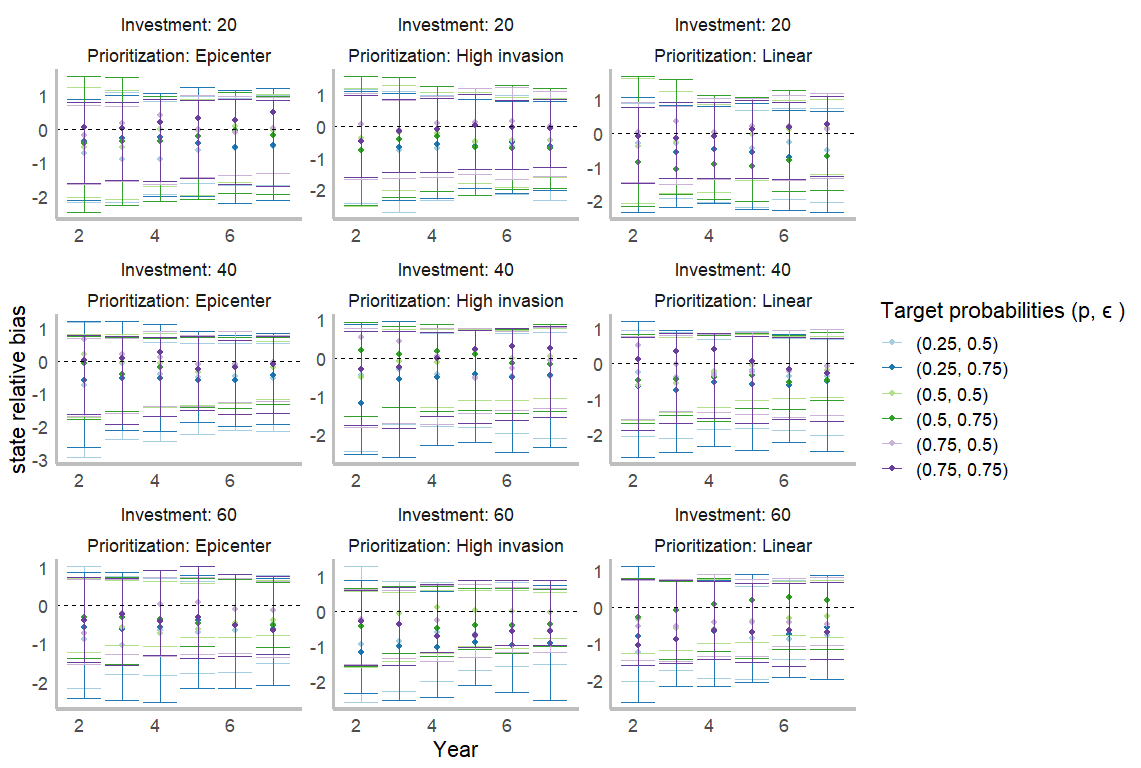


**Figure S6** The change in the relative bias in estimated state through time (not including the first three years in which the alternatives had the same outcomes) for established initial invasion (condition 1).


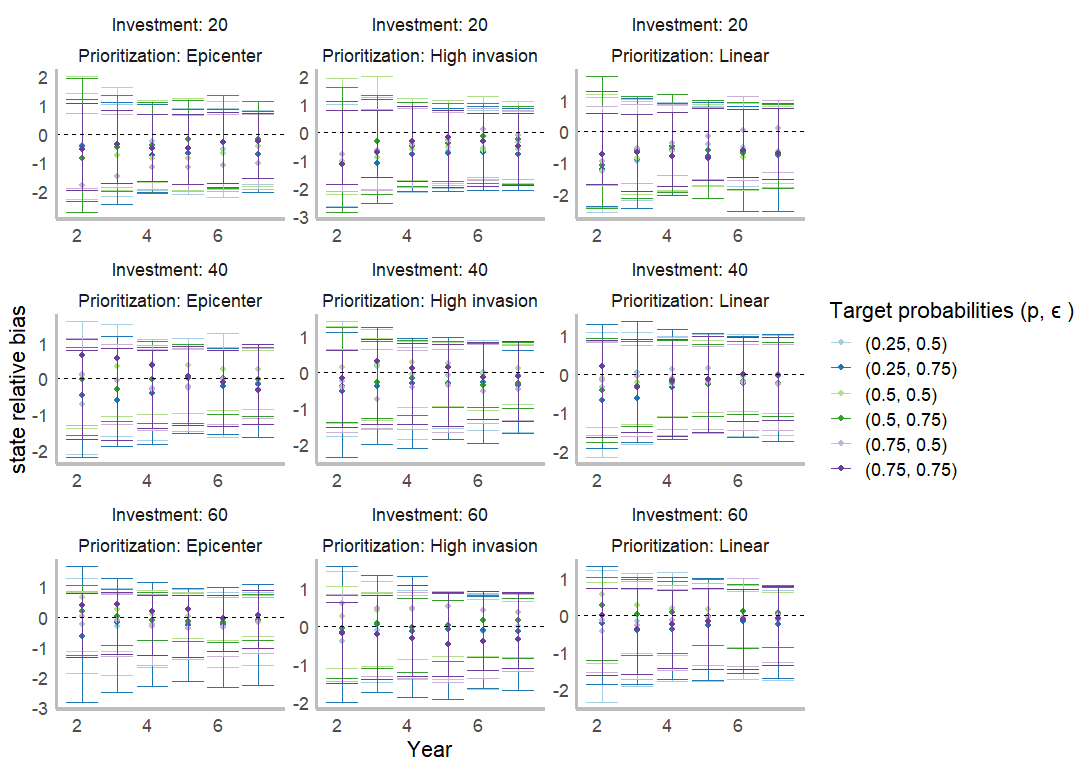


**Figure S7** The change in the relative bias in estimated state through time (not including the first three years in which the alternatives had the same outcomes) for emergent invasion (condition 2).


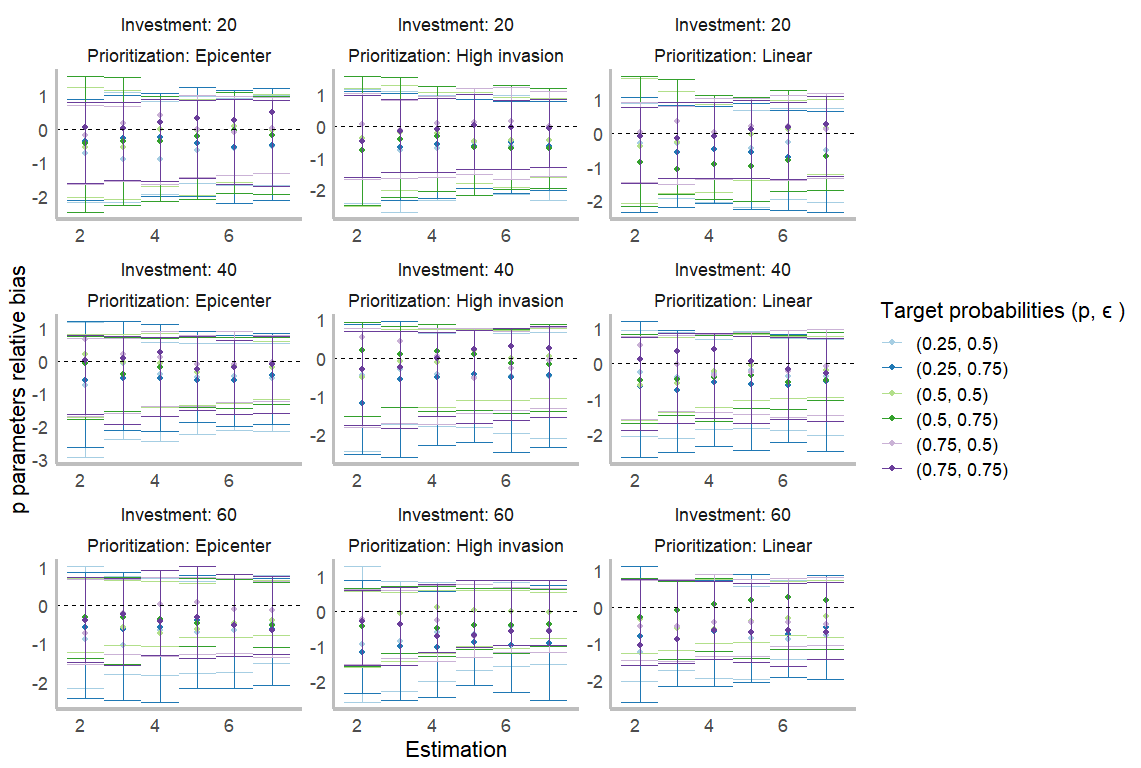


**Figure S8** The change in the relative bias in detection probability, *p*, through time (not including the first three years in which the alternatives had the same outcomes) for established initial invasion (condition 1).


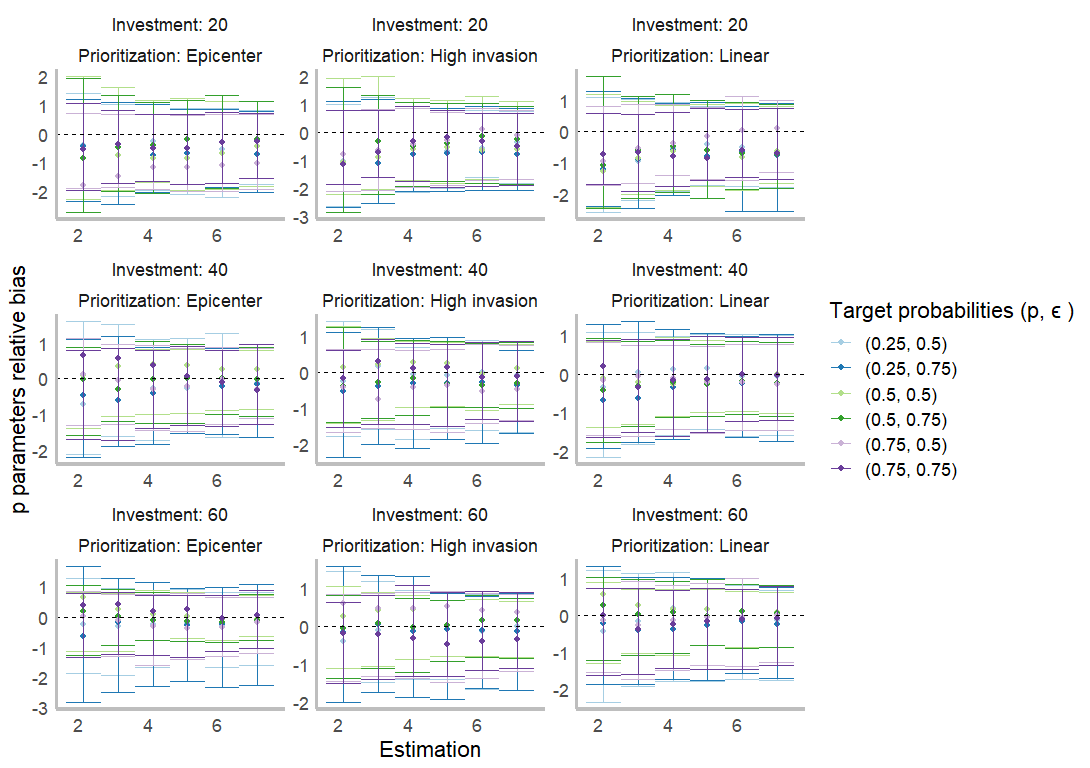


**Figure S9** The change in the relative bias in detection probability, *p*, through time (not including the first three years in which the alternatives had the same outcomes) for emergent initial invasion (condition 2).


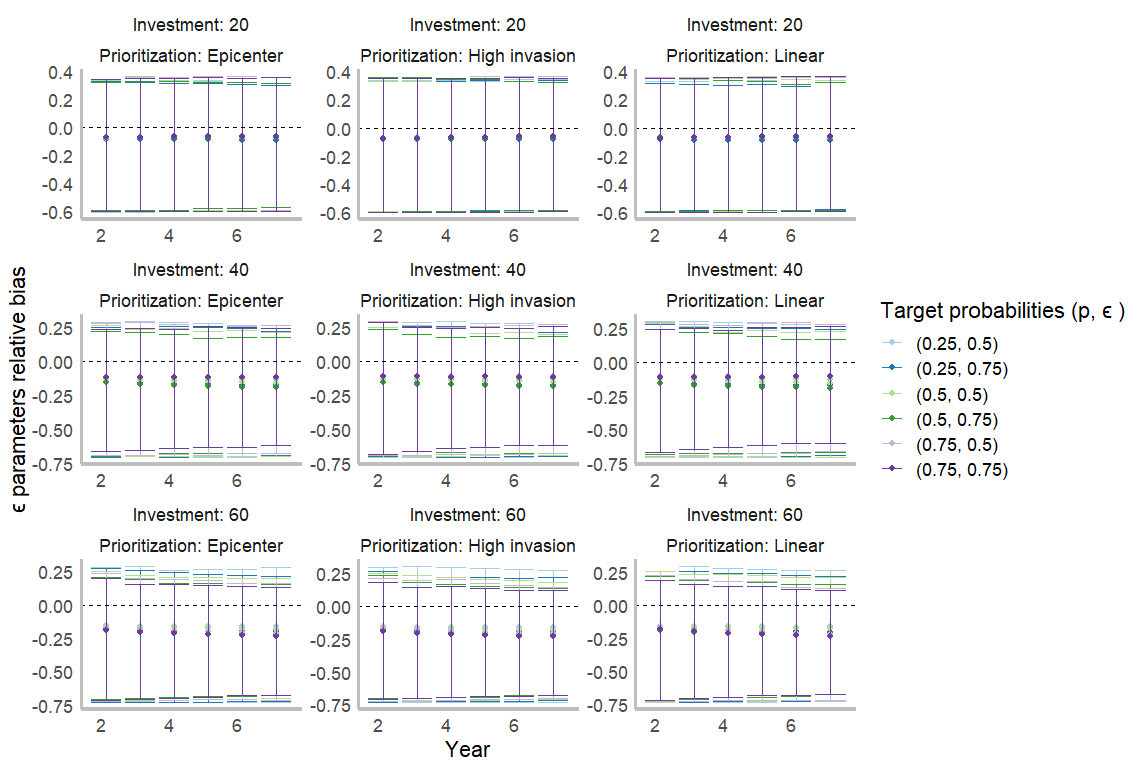


**Figure S10** The change in the relative bias in eradication probability, $\epsilon$, through time (not including the first three years in which the alternatives had the same outcomes) for established initial invasion (condition 1).


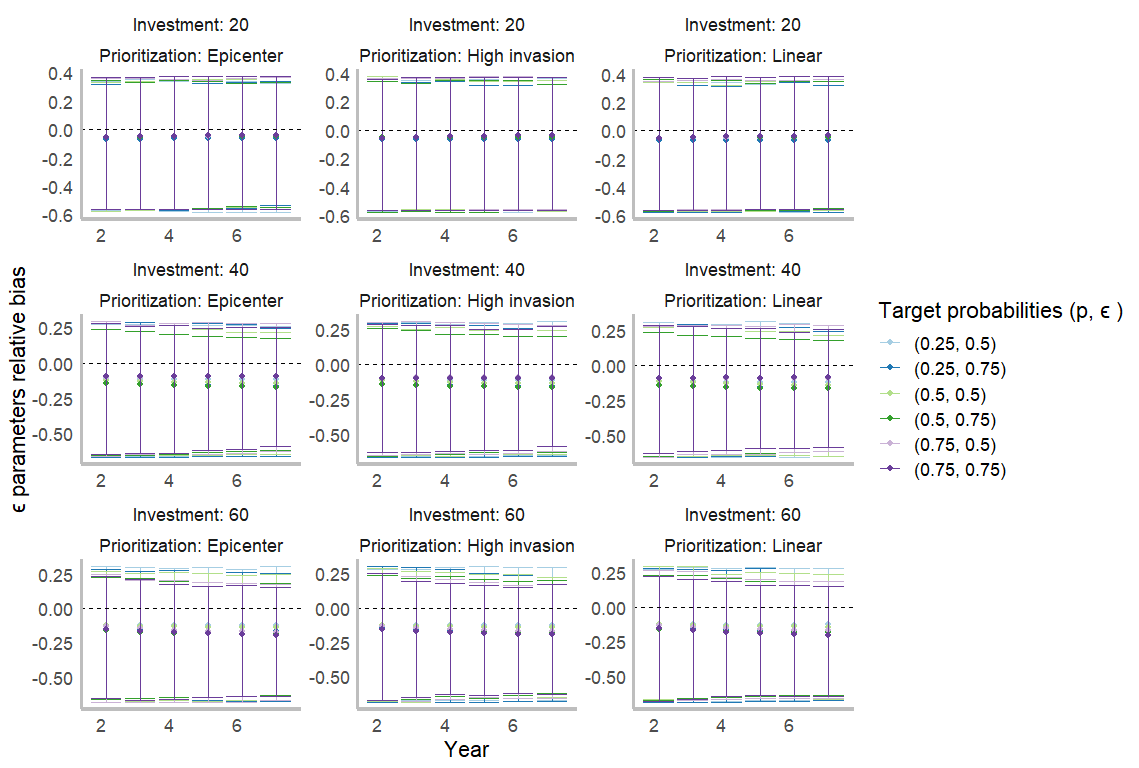


**Figure S11** The change in the relative bias in eradication probability, $\epsilon$, through time (not including the first three years in which the alternatives had the same outcomes) for emergent initial invasion (condition 2).
